# State of the Art Review for Titanium Fluorine Glasses and Glass Ceramics

**DOI:** 10.3390/ma17061403

**Published:** 2024-03-19

**Authors:** Brenna Kettlewell, Daniel Boyd

**Affiliations:** 1 School of Biomedical Engineering, Dalhousie University, Halifax, NS B3H 4R2, Canada; brenna.kettlewell@dal.ca; 2 Department of Applied Oral Sciences, Faculty of Dentistry, Dalhousie University, Halifax, NS B3H 4R2, Canada

**Keywords:** titanium, fluorine, glass, glass ceramic

## Abstract

Titanium (Ti) and fluorine (F) have the potential to provide a variety of desirable physical, chemical, mechanical, and biological properties applicable to a broad range of indications. Consequently, Ti- and F-containing glasses and glass ceramics are currently under investigation for use in nuclear, optical, electrochemical, dental, and industrial fields. Accordingly, significant interest exists with respect to understanding the individual and interaction effects that these elements have on material structure and properties to support the accelerated design, development, and deployment of these materials. This review aims to serve as a foundational reference across multiple disciplines, highlighting the fundamental properties and versatility of Ti- and F-containing glasses and glass ceramics. By consolidating our current knowledge of these materials, this broad overview will identify areas in which we can further our understanding to support the a priori prediction and effective design of these systems. Finally, this paper will introduce the potential to improve material design by integrating experimentation, modelling, and computational approaches in a manner commensurate with the principles of the Materials Genome Initiative.

## 1. Introduction

A major challenge in the design and development of new biomaterials is the complexity associated with attempting to recreate the functions and/or properties of living tissue ex vivo via the incorporation of multiple components (e.g., scaffold, cells, growth factors, and molecular signals) intended to emulate these tissues [1,2]. Despite remarkable discoveries and advances associated with these innovative approaches, the literature contends that this method may, in certain instances, risk over-engineering medical devices, thus limiting their potential translation to clinical use [1]. As a result, an alternative and complimentary design philosophy has emerged in the literature; specifically, it is contended by Place et al. that rather than attempting to engineer complex materials capable of mimicking the intricacy of physiological tissues, we should instead aim to develop synthetic materials that establish key interactions with bodily fluids and cells in ways that unlock the “body’s innate powers of organization and self-repair” [1]. In this regard, new designs for bioactive glasses provide a remarkable opportunity to discover and develop structurally simple yet functionally complex materials capable of promoting a range of desirable materials and host responses [3,4,5]. Bioactive glasses are regarded as robust carrier systems for the controlled and localized delivery of therapeutic metal ions (TMI). These TMIs have been associated with the ability to concurrently adjust the physical and chemical characteristics of glasses while also modulating biological responses, including (but not limited to) antibacterial, anti-inflammatory, osteoinductive, and angiogenic processes [6]. The literature has recently provided excellent reviews on several TMIs [6,7,8]; however, new candidate elements are emerging, and as such, the field is driving exciting new discoveries across an array of applications [9]. Two bioinorganic ions of particular interest for their potential to modulate glass structure and properties, both individually and synergistically, as well as their ability to elicit desirable biological responses, are titanium (Ti) and fluorine (F). 

Ti- and F-containing glasses and glass ceramics are being investigated across an array of scientific and industrial applications. Specifically, and with respect to the effects of Ti on the physical and chemical characteristics of such materials, the literature notes that variations in the coordination and valency of Ti ions may be expected to cause structural modifications and local field variations within glass networks [10,11,12]. According to Sun’s Single Bond Strength Criterion and Dietzel’s Field Strength Criterion, Ti is characterized as an intermediate element [13]. This feature provides the unique opportunity to tailor Ti-containing glasses for a range of indications; for instance, the empty or unfilled *d*-shells of Ti ions may contribute to non-linear polarizabilities, making TiO_2_ substituted glasses promising candidates for use as nonlinear optical devices [14]. Materials consisting of mixed valence state Ti ions are also probable candidates as cathode materials for photoelectrochemical cells (PECs) [15]. Additionally, TiO_2_ polymorphous materials are large bandgap semiconductors (3.2 eV) and, due to their high storage capability, cycling stability, and charging/discharging rate, are considered useful for use as electrode materials for lithium-ion batteries in photocatalysis and in solar cells [15,16]. Furthermore, the addition of Ti to glass and glass-ceramic materials has also been shown to increase chemical durability, thermoluminescent sensitivity, thermal stability, and the mechanical properties of glasses and glass ceramics. As a result, these materials are being explored in the nuclear field for use as thermoluminescent dosimeters [17], immobilization of high-level radioactive waste [18], and gamma-ray shielding materials [19]. 

Contrastingly, the introduction of certain anions, in particular, the halogens and, more specifically, F, into various glass networks has also yielded desirable physical and chemical properties for a range of applications. Fluorine is a network-modifying element and has been shown to replace oxygen ions to form non-bridging oxygens as a result of its similar atomic and ionic radii and electronegativity, thus decreasing network connectivity [20,21]. Fluorine has also been shown to predominantly coordinate with other modifier cations (e.g., Ca, Na), which may lead to the formation of alkali-fluoride-rich regions with enhanced chemical durability [22,23,24]. Thus, understanding the structural role that F may play in various glass networks can inform the design of new materials based on desirable intrinsic (composition and structure) and extrinsic (material performance) parameters. For example, the inclusion of alkaline earth fluorides (e.g., CaF_2_) in borate, phosphate, and silicate glasses has been shown to improve transparency, decrease liquidus temperatures, and reduce melt viscosity [14,25,26]. It has also been established that alkaline earth fluorides produce highly transparent materials and are, therefore, intended as additives for optical materials used in photonics or as scintillators [14]. Additionally, F-containing materials display enhanced electrochemical properties for use in Li-ion cells due to the strong inductive potential of F with phosphates, for example, in lithium fluorophosphate glasses/glass ceramics [27]. As a consequence of their collective impact on the physical and chemical characteristics of glass networks, glasses and glass ceramics containing both Ti and F are of increasing interest to the community, especially with respect to controlling crystallization kinetics, microstructure, and subsequent material properties [19,20,25,26,28,29,30,31]. 

Based on their enhanced crystallization kinetics, Ti- and F-containing glasses and glass ceramics are also being investigated for applications in dentistry, ranging from dental fissure sealants with increased chemical durability [32,33] to ceramic materials for restorative applications and as core materials for veneered resin-bonded ceramic restorations. Accordingly, aside from their ability to modulate specific material properties suited to a range of scientific and industrial applications, Ti- and F-containing glasses and glass ceramics may offer synergies vis-à-vis modulating host responses for biomedical indications. For example, F has been shown to be incorporated into the hydroxyapatite lattice through the substitution of hydroxyl groups [34], leading to the formation of fluoridated apatites (e.g., fluorapatite). Fluorapatite has reduced solubility and increased resistance to erosion due to (i) the densification of the crystal lattice, which is associated with increased acid resistance and (ii) fluorapatite’s lower critical pH (4.5) when compared to that of hydroxyapatite (5.5) [34,35,36,37]. Consequently, F is a highly effective anticaries agent since it can reduce the rates of surface dissolution in enamel, as well as enhance remineralization of teeth [34,38,39]. Additional studies have also established that F is bactericidal, inhibiting the metabolism of dental plaque bacteria responsible for demineralization [39]. Furthermore, F, being both small and the most highly electronegative element, has been investigated for its ability to induce a profound pharmacological effect when bound to carbon in small organic molecules. These effects include improved metabolic stability, altered physiochemical properties, and increased binding affinity of certain compounds such as anti-cancer agents (e.g., thymidylate synthase inhibitors such as 5-fluropyrimidines [40]), antidepressants (e.g., fluoxetine [41]), anti-inflammatory agents (e.g., flufenamic acid and diflunisal [40]), and central nervous system drugs (e.g., sevoflurane, triflupromazine, and fluconazole [42]), to a target protein [43,44]. 

Similarly, the literature contends that Ti may also have potential therapeutic benefits. While the biological role of Ti in humans is not fully understood, there is evidence of its criticality in several biological mechanisms throughout nature. For instance, ascidians are avid accumulators of Ti, which may catalyze, regulate, and stabilize component monomers associated with the synthesis of biological polymers required for tunic formation or wound healing [45]. In recent years, the investigation of natural phenomena such as this has served as a great source of inspiration in the design of synthetic biomaterials [46]. In this case, nature suggests that Ti might assume a role in the construction or repair of soft tissues. Contrastingly, and with respect to hard tissue engineering, in vitro studies have demonstrated that low doses of Ti (~1 ppm) increase osteoblast proliferation and osteoblast phenotype gene expression [47], a feature which may be advantageous in orthopaedics, oral, and maxillofacial applications. Finally, the literature notes that additional biological roles for Ti include deprotonating difficult-to-deprotonate substrates, such as Ti(IV)-bound water molecules [48], due to the powerful Lewis acidity of Ti(IV) as well as the low-potential redox of a Ti^4+^/Ti^3+^ couple. These reactions may be beneficial in a metalloenzyme active site, wherein Ti can take part in a wide range of processes and biochemical reactions, such as electron transfer, substrate recognition/binding, and catalysis [45]. 

Critically, initial reports have demonstrated that Ti may work in synergy with F. For example, in an effort to develop slow-releasing devices for fluoride in dentistry [49], a mechanism for fluoride fixation in enamel has been proposed in the literature in which the fluoride is bound to a polyvalent metal ion in the form of a strong complex. Specifically, McCann [50] examined various metals (Al, Ti, Zr, La, Fe, Be, Sn, Mg, Zn) and discovered that both fluoride uptake and retention may be enhanced when the tooth is pr-treated with any polyvalent metal capable of forming strong fluoride complexes while simultaneously binding to the apatite crystals. Ti ion pre-treatment showed the maximum uptake and retention of F. This, in combination with Ti and its established ability to substitute Ca^2+^ ions in the apatite lattice, forming a Ti phosphate compound, further increases acid resistance [51]. These data suggest that titanium fluoride complexes are beneficial as a topical treatment in dentistry [51,52,53,54].

While both Ti and F have been demonstrated to elicit desirable material and host responses individually, the literature suggests that there is interest in harnessing the benefits of both elements by incorporating them together in a glass network. The objective of this review is to contextualize the impact that Ti and F may have, both individually and synergistically, in modulating glass structure and properties when included together in the composition a glass or contiguous glass ceramics. As such, this document is intended to provide a timely consolidation of our present understanding in this regard. Specifically, this review is intended to provide a state-of-the-art summary of Ti- and F-containing glass and glass ceramic materials currently under investigation for use in a variety of scientific, industrial, and biomedical indications, including optical devices, cathode materials, radiation shielding materials, and dental restorative materials. A crucial first step in the characterization of such novel materials, prior to assessing indication-specific properties (e.g., optical, nuclear, magnetic, electrical characteristics), is gathering information related to the physical and chemical characterization of such materials so as to understand (i) the fundamental role that each element plays in the network and (ii) how that role may vary depending on the chemical composition of the glass/glass ceramic. These physical and chemical characteristics include but are not limited to material composition, extractable and leachable, structural composition or configuration, thermal properties, density and molar volume, chemical durability, size and morphology, topography, surface chemistry, surface energy, bulk characteristics, and mechanical properties. Accordingly, this paper intends to consolidate existing knowledge of the effects that Ti and F have on the physical, chemical, mechanical, and biological characteristics of glasses and glass ceramics containing both elements with varying chemical compositions. A summary of the experimental tests utilized to study these materials and the associated experimental results will be provided to identify areas in which we can further our knowledge and understanding of Ti- and F-containing glasses and glass ceramics. Finally, this paper will introduce the potential to improve glass material design by integrating experimentation, modelling and computational approaches in a manner that strengthens our ability to support the a priori prediction and effective design of new materials with desirable attributes in a manner consistent with the principles of the Materials Genome Initiative.

## 2. Methodology

To clearly establish the potential impact and unique contribution of titanium and fluorine on the physical, chemical, mechanical, and biological properties of glasses and glass ceramics, an initial search strategy was completed using the search strings shown in Table 1. ‘Web of Science’ and ‘PubMed’ databases acted as primary sources for peer-reviewed literature. 

Eligibility of the papers was established in line with the objectives of this work; specifically, the inclusion criteria adhered strictly to (1) glasses and/or glass ceramics that (2) contain both Ti and F and (3) provided confirmation of structure with XRD verification. Papers excluded from this review include those which did not meet the above-mentioned criteria, along with papers investigating slag materials. The associated search results are summarized in Table 2.

## 3. Results

**Figure 1:**
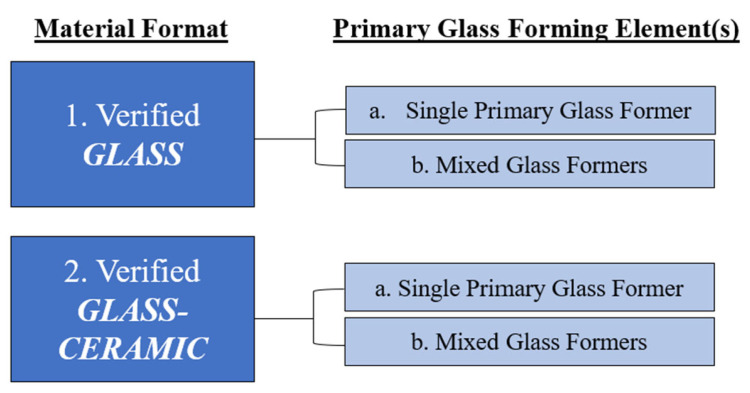
Primary and secondary categories by which each article was categorized.

As presented in Table 2, a total of 3217 articles met the search streams summarized in Table 1. Having hand-reviewed each search result, a total of 26 articles were identified as candidates for inclusion in this work. One of these articles [55] could not be reviewed due to translational barriers, thus warranting its exclusion from the current assessment. The total number of relevant papers was therefore reduced from 26 to 25. In addition to these articles, there were seven [56,57,58,59,60,61,62,63] which classified the material under investigation as a ‘glass’ (6) or ‘glass-ceramic’ (1) but did not provide XRD verification to support this claim. Although these articles met other inclusion criteria, they were excluded from this review based on the lack of the provision of XRD data. The final articles were grouped into categories based on the format and chemical composition of the material being described (Figure 1). 

The material format was divided into (1) verified glasses and (2) verified glass ceramics. A glass can be defined as a “non-crystalline solid exhibiting glass transformation behaviour” or simply as “an amorphous solid” in which the amorphous characteristic is intended to describe atomic disorder as evidenced by an X-ray diffraction (XRD) analysis [13]. Glass ceramics on the other hand can be defined as “inorganic, non-metallic materials prepared by controlled crystallization of glasses via different processing methods” [64]. They contain at least one type of functional crystalline phase, of which the volume fraction may vary from ppm to almost 100%, and a residual glass phase [64]. Accordingly, for the purposes of this review, articles investigating glasses that exhibited signs of crystallinity via XRD analysis but were not deliberately heat treated, were categorized as glasses. Furthermore, articles that studied glass-ceramic materials, but performed experimental tests on both the glass precursor material (prior to heat treatment) and the glass ceramic (after heat treatment), were categorized under glass ceramics. For completeness, the findings derived from testing the glass precursor and those derived from testing the glass ceramic will be discussed separately.

Both material formats were divided and further characterized according to the primary glass-forming element(s) incorporated into each network. Articles investigating glasses/glass-ceramics including only one glass-forming element (e.g., B, Si, P, Ge) in the network were grouped into the (a) single primary glass former category, whereas those that had multiple glass-forming elements (≥2) (e.g., borosilicate, borophosphate, and phosphosilicate) were grouped into the (b) mixed glass formers category. For enhanced clarity, papers that examined multiple glass compositions arising from a baseline glass (i.e., engineered with a single glass-forming element), in particular, those which substituted or otherwise added small amounts of additional network formers [26,30], were grouped based on the baseline glass former.

This paper is structured to review the findings within each of the specified categories based on (1) the materials chemistry as it pertains to the inclusion of Ti and F, and (2) how Ti and F influence the network and material behaviour in terms of physical, chemical, mechanical and biological characteristics. For each article investigating glass materials, specific data such as author, year of publication, area of application, melting process, experimental data collected, and chemical composition has been summarized in Table 3. Table 4 presents the main experimental findings from each article as they relate to Ti and F inclusion, wherein only information related to the scope of this review was included (i.e., the effects of Ti and F on physical, chemical, mechanical, and biological characteristics). Table 5 and Table 6 present the same for articles investigating glass-ceramic materials, respectively. 

**Figure 2:**
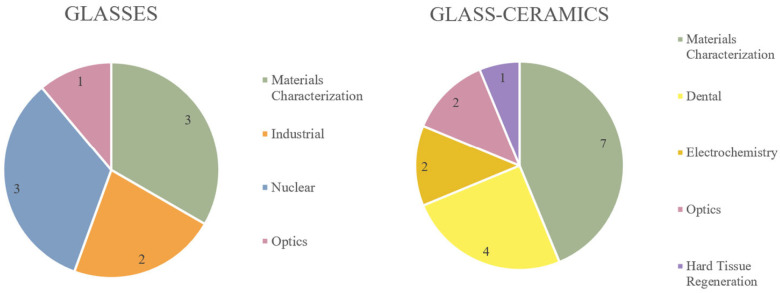
Areas of indication (and number of articles per area) for articles investigating glasses and glass ceramics.

The initial search yielded > 3200 articles, which varied over a wide range of indications, including dentistry, orthopaedics, optics, industrial, electrochemistry, nuclear, physics, and mechanical characterization. As shown in Figure 2, the glass materials were most prominently investigated with respect to generalized materials characterization and nuclear indications, while the most common areas of application for glass ceramics were materials characterization and dentistry. Of the 25 articles under review, the majority characterized phosphate networks (10), followed by networks containing mixed glass formers (Si/P (2), Si/B (2), B/P (2)), borate networks (4), silicate networks (4), and F-only networks (1). 

## 4. Discussion

The objective of this research was to examine the existing literature as it pertains to the inclusion of both Ti and F in glasses and glass ceramics. Having reviewed > 3200 papers and shortlisting 25 based on the inclusion/exclusion criteria described in the methodology section, the authors conclude that the literature is ambiguous and, at times, contradictory with respect to the effects that Ti and F have on the physical, chemical, mechanical and biological properties of these materials. For clarity, this discussion will be structured such that the findings related to glass materials are discussed first, followed by those related to glass-ceramic materials. 

### 4.1. Glasses

The observed trends (increasing or decreasing) in a selection of experimental findings associated with an increasing content of Ti and/or F in glass materials are presented in Table 7. There are multiple categories in which the literature indicates contrasting trends with respect to the effects of Ti and F on glass structure and properties. Although these contrasting trends are primarily a result of varying glass chemistries, contradicting results exist between glasses with similar compositions. Furthermore, there are several properties where the effects of increasing Ti and F content have not yet been addressed, highlighting large gaps in our knowledge of Ti- and F-containing glass and glass ceramics despite their significant potential across a broad range of applications. 

#### 4.1.1. Coordination Number

As a result of overall glass chemistry, the literature indicates contrasting trends with respect to the influence of Ti on the coordination number of glass-forming elements. For instance, an increase in coordination number (upon the addition of Ti) occurred in networks where the primary glass-forming system was phosphate [18] or borosilicate [19]. Mechanistically, it has been determined from Fourier transform infrared spectroscopy (FTIR), Raman spectroscopy, and nuclear magnetic resonance (NMR) that Ti may act as a network former in such systems. Specifically in phosphate glass, the addition of TiO_2_ altered the phosphate network by breaking P=O bonds to form P-O-Ti bonds, which increased cross-linking and strengthened the phosphate chains [18]. For the borosilicate glass, the average coordination number and number of bridging oxygens increased as ZnO was substituted by TiO_2_. Therefore, as the content of TiO_2_ increased, there was an increase in interconnection [19]. 

Contrastingly, the coordination number of the network former decreased with increasing Ti in networks formed exclusively with boron as the glass former. This decreasing trend appears to be consistent in the literature. However, there are only three articles [14,65,66] that provided data on the effects of Ti vis-à-vis network coordination numbers in borate glasses, two investigating the same glass system [65,66]. It was reported that low concentrations of TiO_2_ (where the Na_3_AlF_6_ modifier behaviour was predominant [65]) were associated with the presence of TiO_4_ units and appeared to promote an increased population of B4 units in borate glasses. Contrastingly, high concentrations of TiO_2_ were associated with the presence of TiO_6_^2−^ units and increased the population of B3 units [65]. The conversion of B4 to B3 observed in TiO_2_-rich glasses was likely associated with the Ti coordination change as a result of TiO_4/2_ + 2B(O,F)_4_ → Ti(O,F)_6_ + 2BO_3_ [65,66]. In a separate borate glass network [14], data derived from FTIR show that as the concentration of TiO_2_ increased in the glass samples, the intensity of the bands attributed to TiO_6_ and BO_3_ increased, while the bands attributed to TiO_4_ and BO_4_ decreased in intensity [14]. Although the decreasing trend in coordination number upon the gradual addition of Ti appears to be consistent across these three articles, there are contrasting findings as it relates to the concentration of Ti. Specifically, Anghel, Florian and Bessada found that low concentrations of TiO_2_ (0–4.4 wt.%/0–10.54 mol%) were associated with an increased presence of B4 units, whereas Lakshmi and Cole reported that TiO_2_ (0–0.7 mol%) acted as a modifier, increasing the presence of B3 units. Existing literature is conflicted with respect to the role of Ti in the structural modification of glasses. Like many other areas of glass science, this may be due to the individual effects of Ti on the network or, more likely, the interaction effects between Ti and other components. Describing a mechanism by which the interaction effects of network constituents influence material properties will require the development of new experimental approaches with the capacity to identify individual and interaction effects. Further research is necessary to elucidate this role.

Two articles [65,66], both investigating networks in which boron was the primary network-forming element, provided data relating to the effects of F on coordination number. It was observed that increasing the content of F in the network led to a decrease in the coordination amount of boron. In samples with low TiO_2_ content (e.g., 80T11), the lower concentration of high field strength Ti^4+^ cations resulted in increased fluorine uptake by boron. This allowed F^−^ ions to substitute O^2−^ ions, leading to the formation of non-bridging oxygens and causing the conversion of B4 units to B3 units [66]. While the findings from both articles are consistent, it is important to note that they share certain limitations. Firstly, both articles studied the same glass compositions and were authored by the same researchers. This lack of variability in the data sources restricts our ability to make accurate predictions about the effects of F (fluorine) across a wide range of concentrations on the structure of glasses with different chemical compositions. To gain a more comprehensive understanding of the impact of fluorine on glass structures, it is crucial to conduct studies encompassing a broader range of glass chemistries. Such investigations should be structured to uncover the nuanced interactions between fluorine and various glass components, allowing for more generalized and robust conclusions. By exploring a wider spectrum of glass compositions, the literature may better establish how fluorine influences glass properties under different chemical scenarios, leading to a more thorough comprehension of its effects on glass structures.

#### 4.1.2. Density and Network Connectivity

The literature indicates varying trends with respect to the effects of increasing Ti content on the density and network connectivity of Ti- and F-containing glasses. For instance, increased density and network connectivity (upon the addition of Ti) occurred in networks where the primary glass-forming system was borophosphate [16], phosphate [18], and borosilicate [19]. Specifically in phosphate and borophosphate glasses, the addition of TiO_2_ has been shown to strengthen the network by creating cross-links between phosphate and borate chains (P-O-P and B-O-B linkages are substituted by P-O-Ti or B-O-Ti linkages), thus increasing the bulk density [16,18]. Similarly, an increase in the number of bonds per unit area and the cross-linking density was observed with increasing Ti content in borosilicate glasses [19]. 

Contrastingly, the density and network connectivity decreased with increasing Ti in networks formed exclusively with boron. In one borate glass system [14], density decreased with increasing TiO_2_ concentration because zinc fluoride was gradually substituted by titanium oxide, which has a comparatively lower density and lighter atomic weight than zinc fluoride [14]. Additionally, it was reported that the addition of glass modifiers, such as Ti^4+^, into borate networks, may cause an increase in the number of non-bridging oxygen attached to large ring borate groups (e.g., diborate rings, pentaborate rings, di-pentaborate rings, and triborate rings), thus disrupting these ring structures and decreasing the connectivity of the network [65]. This resulted in the formation of smaller non-ring borate groups (e.g., diborate, triborate, and pentaborate) in the network [65]. The findings derived from the literature [14,18,19,65] agree with the reported trends in coordination numbers summarized above, wherein a decrease in coordination number is often associated with a decrease in network connectivity. Only two articles [15,16] reported the effects of Ti addition on the density and network connectivity of a glass with the same glass-forming system, i.e., borophosphate. Contrary to the findings of Guntu et al. [16] discussed above, Rua and Kumar [15] found that an increase in TiO_2_ content caused an increase in non-bridging oxygens, which induced higher grades of disorder within borophosphate networks and resulted in a decreased network connectivity. These two articles [15,16] observed opposing and contrasting results, which emphasize the difficulties associated with predicting the effects of Ti and F inclusion in certain glass systems. 

#### 4.1.3. Glass Transition Temperature

The increasing content of TiO_2_ in phosphate and borosilicate glass networks has been shown, by means of differential thermal analysis (DTA), to increase glass transition temperature (T_g_) [18,19]. Specifically, the thermal stability of Ti-containing phosphate glasses was greater than that of a Ti-free glass sample because the addition of TiO_2_ was shown to strengthen the network by creating cross-links between phosphate chains [18]. Similarly, in borosilicate glass, as the content of TiO_2_ increased, more bridging oxygens formed and there was an increase in interconnectivity of the network [19]. This increase in connectivity is regarded as the mechanism underlying the observed increase in T_g_ and is consistent with the observations noted with respect to coordination number, density, and network connectivity in these systems. 

In juxtaposition to phosphate and borosilicate glasses, the addition of TiO_2_ may cause minor decreases in the T_g_ in borophosphate glass [15]. Specifically, Rao and Kumar reported that increasing the content of TiO_2_ up to 0.6 mol% led to a reduction in T_g_ from 445 to 440 °C. Mechanistically, the decrease in T_g_ was believed to be a result of the increased number of non-bridging oxygens and decreased network connectivity associated with the addition of TiO_2_ to the network [15]. The authors further report that at increased addition of TiO_2_ (at 0.8 mol% and 1 mol%), the glass transition values began to increase to 441 and 442 °C, respectively [15]. However, the observed decrease in T_g_ may be within the error of the DTA equipment, and so careful consideration should be given to this dataset. Furthermore, the T_g_ has been shown to decrease upon the addition of TiO_2_ to borate glass [17]. Regrettably, however, this specific article provided DTA curves for only two glass chemistries: LBA (50Li_2_O·45B_2_O_3_·5Al_2_O_3_) and LBA·50LiF_2_·xTiO_2_ (wt.%). Therefore, the decrease in T_g_ observed in the LBA·50LiF_2_·xTiO_2_ glass compared to the LBA glass could have been due to the added LiF_2_ (or a combination of the added LiF_2_ and TiO_2_) rather than solely the TiO_2_. Consequently, this article was excluded from Table 7. To prevent these ambiguities in future research, we should aim to gather baseline data on the effects of gradual Ti and F addition across broad composition ranges using a systematic approach capable of predicting the effects of multiple constituents within a network.

#### 4.1.4. Mechanical Characteristics

There was one article that evaluated the effect of TiO_2_ addition on the mechanical properties of Ti- and F-containing glasses [16]. As CaF_2_ was increasingly substituted by TiO_2_ in a borophosphate glass, Young’s modulus, shear modulus, and bulk modulus were all observed to increase, while the microhardness and Poisson’s ratio decreased [16]. The substitution of divalent Ca^2+^ ions by Ti^4+^ ions, as well as the substitution of P-O-P and B-O-B bonds for stronger P-O-Ti and/or B-O-Ti bonds upon the addition of TiO_2_, led to an increased packing density within the glasses. The increased packing density, also associated with increased material rigidity, was reported to have led to an increase in the various elastic moduli. Additionally, the observed trends in Poisson’s ratio and microhardness upon increasing concentration of TiO_2_ suggested that the prepared glasses exhibited an increasingly interconnected structure [16]. Surprisingly, little information is available in the literature on the mechanical properties of Ti- and F-containing glasses, suggesting that further research in this area would be of considerable value to the community.

### 4.2. Glass Ceramics

The observed trends in a selection of experimental findings associated with increasing content of Ti and/or F in glass-ceramic materials are listed in Table 8. Similar to the findings summarized in Table 7 for glass materials, the literature indicates varying trends with respect to the effects of Ti and F on glass-ceramic structure and properties, depending on glass chemistry. Additionally, as shown in Table 8, there are properties for which the effects of Ti and F addition have not yet been addressed in the literature. For a selection of the findings listed in Table 8, the experimental test was performed on the glass-ceramic precursor material (the glass intended to produce a glass ceramic) prior to heat treatment. These results are denoted with an asterisk in Table 8. The following section will be structured to discuss the findings of tests that were performed on the glass-ceramic precursors, followed by those that were performed on the glass ceramics. 

#### 4.2.1. Glass-Ceramic Precursor

The findings discussed in the following section are derived from experimental tests that were performed on glass-ceramic precursor materials prior to heat treatment.

##### Coordination Number

Among the 16 articles investigating glass-ceramic materials, one article examined the coordination number of a glass precursor material before heat treatment [26]. In that study, CaF_2_ was added to a Ti-containing glass with silica as the network-forming element, leading to a decrease in the coordination number of silica [26]. Raman bands from the glass-ceramic precursor were attributed to Si-O stretch vibrations of Qn (*n* = 1, 2, 3, 4) tetrahedral units. The addition of CaF_2_ caused a reduction in the fraction of Q4 units, indicating disruption of the SiO_2_ three-dimensional network. Specifically, F^−^ ions replaced bridging oxygens in =Si-O-Si= with weak =Si-F linkages, weakening the glass network [26]. Since the decrease in coordination number was observed only for the specific compositions T and TF, a continuous trend in coordination number upon the gradual addition of F could not be established. To achieve a deep scientific understanding of fluorine’s influence on glass structures, it is imperative to undertake studies involving a wider array of glass chemistries. Embracing diverse investigations will unveil the intricate interactions between fluorine and various glass components, facilitating more comprehensive and robust evaluations of such materials. By delving into a broader spectrum of glass compositions, we may effectively evaluate how fluorine impacts glass properties within distinct chemical scenarios, fostering a deeper comprehension of its effects on glass structures. Indeed, addressing this complex challenge can be achieved through the enhanced utilization of the design of mixture methodologies and the integration of machine learning techniques. By combining these powerful tools, we can accelerate the pace of research and facilitate a more rapid and comprehensive development of our understanding in this area. The design of mixture methodologies allows for the systematic exploration of a wide range of glass compositions, optimizing the experimental design for efficient data collection. Coupling this with machine learning enables the extraction of valuable patterns and trends from large datasets, guiding researchers towards novel insights and accelerating the discovery process. This synergistic approach holds the potential to revolutionize glass science and propel our understanding of fluorine’s impact on glass structures to new heights.

##### Glass Transition Temperature

The literature indicates that increasing Ti content has contrasting effects on the T_g_ of Ti- and F-containing glasses, depending on glass chemistry. An increase in T_g_ has been reported to occur with increasing Ti content in networks where the primary glass-forming system is borosilicate [31] or silicate [72]. It was found that by increasing the TiO_2_ content from 1–10 wt.% in a borosilicate network, the T_g_ and both the crystallization peak temperatures increased (T_g_: 705–723 °C, T_p_^I^: 874–902 °C, T_p_^II^: 938–980 °C) [31]. The increase in these thermal characteristics was believed to be due to the high field strength of the Ti^4+^ ion, which accelerates the interaction between structural units and results in the formation of linkages between some positive cations, such as Mg^+^ and K^+^. This finding is consistent with the increased T_g_ observed by Shaaban et al. upon the addition of TiO_2_ in a borosilicate glass [19]. Furthermore, the addition of TiO_2_ to a glass formed exclusively with silicate significantly increased the glass transition and dilatometric softening point temperatures [72]. While the exact mechanism behind this increase in T_g_ was not discussed, Takav et al. [72] concluded that the addition of TiO_2_ to the base composition increased the glass viscosity, thus decreasing the occurrence of crystallization.

In contrast to the above-noted findings, a decrease in T_g_ was associated with the addition of TiO_2_ in networks where the primary glass-forming system was phosphosilicate [73]. Mechanistically, the decreased T_g_ was attributed to the decrease in viscosity that came from gradually adding Ti into the network. It was reported that the addition of Ti^4+^ ions accelerated the formation of cross-links between phosphate units, thus disrupting the glass network and decreasing the glass viscosity [73]. Additionally, the T_g_ of a phosphate glass was found to decrease upon the addition of TiO_2_ [27]. Specifically, when comparing the T_g_ of the VV sample (Li_3_V_2_(PO_4_)_2_F_3_) to the TV sample (Li_3_TiV(PO_4_)_2_F_3_), that of the TV sample was lower. Because this finding was derived from comparing the T_g_ of only two different glass compositions (VV and TV), a continuously decreasing trend in T_g_ upon the gradual addition of Ti could not be identified. To provide a more comprehensive understanding of the effects of Ti on the T_g_ of glasses, multiple compositions of varying Ti content should be investigated. It can be concluded, however, that the addition of TiO_2_ to the phosphate glass caused an overall decrease in T_g_, contrary to the results found for the phosphate glass previously described by Lu et al. [18]. 

The addition of F was found to decrease the T_g_ in both silicate [26] and phosphate [33] glasses. In silicate glass networks, T_g_ and T_p_ were both found to decrease upon the addition of CaF_2_ [26]. With the addition of CaF_2_, F^−^ ions substituted the bridging oxygens in =Si-O-Si= due to the similar radius between oxygen and fluorine. The replacement of the strong linkage with a pair of non-bridging =Si-F linkages caused a decrease in viscosity, an increased occurrence of crystallization, and a decreased T_g_ and T_p_ [26]. The changes that were observed in these parameters upon adding CaF_2_ were also hypothesized to be due to phase separation in the melts. Specifically, fluorides can be immiscible in silicate melts, resulting in nucleated droplet phase separation that facilitates the occurrence of crystallization [26]. Because the decrease in T_g_ was observed only by comparing two compositions (T and TF), a continuous trend in T_g_ upon the gradual addition of F could not be identified. Thus, to provide a more comprehensive understanding of the effects of F on the T_g_ of glasses, multiple compositions of varying F content should be investigated. Separately, the inclusion of F also resulted in a decreased T_g_ in phosphate glasses [33]. The mechanism behind this decrease in T_g_ was not discussed. This presents an excellent opportunity to contribute new knowledge to the Ti- and F-containing glass literature regarding the effect of F on the T_g_ of phosphate glasses.

##### Mechanical Characteristics

As mentioned above, it was found that the addition of CaF_2_ to silicate glass caused a decrease in glass viscosity [26]. Mechanistically, F^−^ acted as a network modifier, weakening the glass structure and replacing the bridging oxygens in =Si-O-Si= due to the similar radius between oxygen and fluorine. Consequently, the formation of weak =Si-F bonds led to the weakening of the polymerization of the glass network and, thus, a decrease in viscosity.

#### 4.2.2. Glass Ceramics

The following section presents findings derived from experimental tests on glass-ceramic materials after undergoing heat treatment.

##### Coordination Number

In the literature, the influence of titanium (Ti) on the coordination number of the primary glass-forming element(s) in Ti- and F-containing glass ceramics has not been explicitly discussed. However, insights from chemical durability and Raman analyses in a phosphate glass [25] reveal valuable information. It was observed that TiO_2_ concentrations of up to 0.6 mol% led to a preference for tetrahedral configurations of Ti ions, promoting their active involvement in network formation with increased P-O-Ti bonds in the glass network. Simultaneously, up to 0.6 mol% TiO_2_ addition, the Raman bands linked to TiO_6_ units showed a decrease in intensity. Conversely, when the TiO_2_ concentration was raised from 0.6 to 0.8 mol%, a different trend emerged. The Raman intensity associated with TiO_6_ structural units increased, while that of TiO_4_ structural units decreased. These results suggest that within the range of 0.6–0.8 mol% TiO_2_, Ti ions predominantly formed octahedral configurations, which, akin to Ti^3+^ ions, may participate in the formation of non-bridging oxygens. Though not explicitly discussed in the article, based on these findings, it can be inferred that Ti addition up to 0.6 mol% enhanced the phosphate coordination number of this glass system, while further addition of TiO_2_ up to 0.8 mol% decreased the coordination number.

##### Density

A single article examined the impact of Ti addition on the density and network connectivity of glass ceramics [25]. The introduction of TiO_2_ (up to 0.6 mol%) in a phosphate glass ceramic resulted in an increased density, while subsequent increases in TiO_2_ (from 0.6 to 0.8 mol%) were associated with a slight decrease in density. The progressive increase in density up to 0.6 mol% TiO_2_ indicated a growing structural compactness in the material. This can be attributed to the greater presence of Ti ions in a Ti^4+^ state, as confirmed by Raman spectroscopy [25]. Notably, up to 0.6 mol% concentration, Ti ions exhibited a preference for tetrahedral positions, fostering more P-O-Ti bonds rather than acting as modifiers that create additional non-bridging oxygen bonds in the glass network. Furthermore, because Ti ions in the Ti^4+^ state possess a higher field strength compared to the Ti^3+^ state, thus leading to an overall increase in material compactness, the authors suggested that the increased density up to 0.6 mol% TiO_2_ was an indication of Ti ions existing primarily in the Ti^4+^ state in sample T6 (0.6 mol% TiO_2_).

##### Glass Transition Temperature

The introduction of Ti into glass-ceramic networks, where the primary glass-forming system is phosphate, has been found to result in an increase in the glass transition temperature (T_g_) [25]. Particularly, it was observed that as the concentration of TiO_2_ was raised up to 0.6 mol%, there was a corresponding increase in T_g_ [25]. This rise in T_g_ can be attributed to the augmented cross-link density and closer packing facilitated by the increasing presence of tetrahedral Ti ions, as discussed in the preceding section [25].

##### Mechanical Characteristics

In the study of glass ceramics, the evaluation of mechanical properties was more prevalent compared to glasses. Table 8 presents the ambiguities and contrasting trends in various characteristics, such as microhardness and viscosity, in relation to the addition of TiO_2_ to glass ceramics. For instance, the incorporation of TiO_2_ into a silicate glass ceramic was found to result in an increase in the glass viscosity [72]. Consequently, the glass ceramics containing TiO_2_ experienced less interruption from crystallization, leading to improved sinterability. This, coupled with the observation of harder crystalline phases precipitating within the material’s microstructure, contributed to an increase in microhardness with the progressive addition of TiO_2_ [72]. However, it is important to note that the increased TiO_2_ content also resulted in suppressed crystallinity and a reduced interlocking crystalline arrangement. Consequently, this caused a decrease in fracture toughness and flexural strength in glass ceramics [72].

In a phosphate glass ceramics, it was found that the gradual increase in Ti content (up to 0.6 mol%) caused an increase in Young’s modulus (i.e., 58–62.6 GPa), shear modulus (i.e., 26.1–28.11 GPa), Poisson’s ratio (i.e., 0.111–0.114), and microhardness (i.e., 6.65–7.24 GPa) [25]. Upon the further addition of TiO_2_ (from 0.6 to 0.8 mol%), there was a decrease in these properties (i.e., 57.2 GPa, 25.7 GPa, 0.113, 6.63 GPa, respectively). This behaviour corresponds well with the density findings reported in the literature [25], wherein the increased density values were indicative of increasing structural compactness and decreasing disorder in the glass network. In support of the above findings, the literature notes that in a less disordered framework, the mechanical loss factor or the coefficient of internal friction of piezoelectric composite oscillators is decreased, leading to an increase in elastic coefficients and micro-hardness of glass materials [25].

Contrary to the increased viscosity observed in silicate glass ceramics with increasing Ti content [72], the viscosity of a phosphosilicate glass ceramic decreased with increasing TiO_2_ [73]. As previously mentioned, this behaviour was reported to be a result of TiO_2_ disrupting the glass network and decreasing glass solubility by forming cross-linking Ti^4+^ units [73,75]. Furthermore, in contrast to the increased microhardness in silicate glass ceramics with increasing Ti content [72], the microhardness of a borosilicate glass ceramic decreased with the addition of Ti [31]. The decreased hardness was believed to be correlated to the formation of the interlocking, dense, blocky microstructure, as evidenced by SEM [31].

##### Chemical Durability

Although chemical durability/dissolution behaviour was not evaluated in any of the articles investigating glasses, there were three articles that studied this behaviour in phosphate [25] and silicate [30,72] glass ceramics. For instance, in phosphate glass ceramics, the addition of TiO_2_ (up to 0.6 mol%) caused an increase in chemical durability of the glass ceramic, whereafter further increase in TiO_2_ (from 0.6 to 0.8 mol%) reportedly led to a slight decrease in chemical durability [25]. The average dissociation rate (DR) of the TiO_2_-free glass ceramic was 5.37 ((×10^−6^) g/cm^2^/min). With increasing content of TiO_2_ up to 0.6 mol%, the DR decreased to 0.81 ((×10^−6^) g/cm^2^/min), whereafter it increased to 1.72 ((×10^−6^) g/cm^2^/min) upon the addition of 0.8 mol% TiO_2_. These results suggested that the addition of TiO_2_ (up to 0.6 mol%) increased the chemical durability of the glass ceramics. This was believed to be due to the titanium ions forming preferentially into tetrahedral configurations, thus participating in the formation of P-O-Ti bonds in the network rather than forming non-bridging oxygens. The addition of TiO_2_ had a similar effect on a silicate glass ceramic [30], wherein the composition that included Ti had a higher chemical resistance compared to the composition without Ti. Unfortunately, only two of the four compositions in the study were investigated for their chemical durability. As a result, observing the effects of a gradual increase in Ti content across multiple compositions was not possible, and a continuous trend in chemical durability could not be derived. Furthermore, the increased chemical resistance of the Ti-containing material cannot be fully attributed to the Ti addition, as the comparative glass compositions varied in phosphate content. Consequently, this result was excluded from Table 8. This example is representative of a fundamental challenge in the design of glass networks. In particular, given the almost limitless compositional arrangements that are possible [76], it is fundamentally clear that alternative design strategies be implemented as soon as possible—so as to elucidate the individual and interaction effects of multiple glass constituents on the composition–structure–property relationships within a glass network [77].

In a separate silicate glass-ceramic system, the gradual increase in Ti content was found to have varying effects on the chemical durability of the material [72]. For instance, there was significantly decreased chemical durability for glass-ceramic FC6 (6 wt. ratio TiO_2_) compared to FC0 (0 wt. ratio TiO_2_), but further increases in TiO_2_ leads to the increase in chemical durability of the glass ceramics FC9 (9 wt. ratio TiO_2_) and FC12 (12 wt. ratio TiO_2_). It was concluded that glass structure connectivity was improved by the increase in TiO_2_ content, which, in turn, resulted in increased chemical stability in the residual glass phase. Presumably, the increased chemical stability of the residual glass phase was responsible for the decreased chemical solubility of FC9 and F12.

There were two articles that reported findings on the influence of F on the chemical durability/dissolution properties of Ti- and F-containing glass ceramics [32,33]. Both articles were written by the same primary author and evaluated very similar phosphate glass networks. From analyzing SEM micrographs of the CaF_2_-containing (CTP-F) and CaF_2_-free (CTP) glass-ceramic surfaces after etching with acid, it was found that the chemical durability of the CaF_2_-containing glass ceramic was drastically improved. Specifically, SEM micrographs showed almost no surface alteration in the CTP-F glass ceramic, whereas the CTP glass-ceramic surface was severely etched. The increased chemical durability was attributed to the addition of F, which induced the preferential formation of apatite crystal throughout the network. It was believed that a large number of orthophosphate groups in the glass were used for the formation of these apatite crystals, causing the amount of CaTi_4_(PO_4_)_6_ crystal formed in the glass to decrease. As a result, the residual Ti constituent resided in the glassy phase of the material. Succinctly, the excellent chemical durability of the phosphate glass ceramics was suggested to originate from the microstructure of the glass-ceramic, specifically, the increased crystalline phases and a high content of Ti in the residual glassy phase [32,33].

## 5. Future Directions

Ti- and F-containing glasses and glass-ceramic materials have applications in industries aimed at addressing challenges in human welfare, national security, and clean energy, yet the current state of the literature confounds our understanding of these materials, specifically with respect to the effects of Ti and F on material structure and properties. Consequently, we are unable to accurately predict the roles of Ti and F (both individually and interactively) in modulating the physical, chemical, mechanical, and biological characteristics of glasses and glass ceramics, hindering the accelerated development of these materials and subsequently delaying their translation to commercial use. Accordingly, further investigation of Ti- and F-containing glasses and glass ceramics is required to support the streamlined discovery, design, and deployment of these materials.

The results summarized in Table 7 and Table 8 reflect the conflicts and gaps in our knowledge relating to the individual roles of Ti and F in glass networks and how they influence various characteristics, such as coordination number, T_g_, density and network connectivity, chemical durability, mechanical characteristics, and biological responses. More critically, the literature contains many assumptions on the role that individual elements play on material properties; however, these analyses are absent crucial considerations relating to interaction effects between elements. Specifically, for many of the experimental findings summarized in Table 7 and Table 8, the literature indicates conflicting observations with respect to the influence that increasing Ti or F content has on glass structure and properties. These contrasting results were mostly a result of varying chemical composition; however, opposing trends were identified for glasses/glass ceramics with the same primary constituents. These contradictions make it extremely difficult to anticipate, predict, and optimize the roles of glass constituents (e.g., Ti and F) for a given indication. In cases where there were consistent trends in the effects of Ti and/or F addition in glasses/glass ceramics with the same glass-forming system, the number of articles reporting these results was very few (≤3). Accordingly, there is a lack of consistency and repeatability in the current literature, making it difficult to accurately predict the influence that Ti and/or F may have on glasses and glass ceramics of varying chemical compositions.

Furthermore, for each experimental finding summarized in Table 7 and Table 8, the trends were often only characterized for a limited number of glass chemistries. For example, the glass networks most evaluated were those where the primary glass-forming system was boron or borophosphate. There were no articles that reported findings on the effects of Ti addition on the properties of oxyfluoride silicate glasses. Alternatively, the glass-ceramic networks most evaluated were those where the primary glass-forming system was silicate or phosphate. There were no articles that reported findings on the effects of Ti addition on the properties of F-containing borate glass ceramics. Moreover, the only networks investigated for the effects of F addition (in glasses and glass ceramics) were borate, silicate, and phosphate. Regrettably, the lack of complete data on a range of alternative glass chemistries (e.g., borate, phosphate, silicate, germanium, phosphosilicate, borophosphate, borosilicate) limits our understanding of the effects that Ti and F may have on the physical, chemical, mechanical, and biological properties of a variety of networks. To address this limitation, we should aim to gather baseline data on the composition–structure–property relationships that exist within varying glass chemistries upon the incorporation of Ti and F across wide compositional ranges.

Additionally, there are notable gaps in the types of responses investigated in these reports. For instance, the effect of F on the density and network connectivity, T_g_, and mechanical characteristics of Ti- and F-containing glasses was not addressed. For glass-ceramic materials, the effect of F on the density and/or network connectivity was not addressed. Furthermore, the chemical durability/dissolution properties of Ti- and F-containing glasses have not been discussed in the literature, meaning that our ability to predict and control this critical aspect of materials design is highly constrained. The evaluation of glass dissolution behaviour provides insight into the glass structure and how it controls which ions are more readily released into the solution. This information is critical in predicting and understanding the rate and mechanism by which the glass will degrade—a process that must be controlled for a variety of applications, from short-term and long-term medical materials to solar cells. With respect to these materials in medicine, there is a substantial lack of knowledge related to the biological safety and efficacy associated with Ti- and F-containing glasses and glass ceramics. Specifically, there is no information available on the characterization of these materials as they relate to host responses, such as mineralization potential and effect on cellular responses (e.g., angiogenesis, cell viability, antimicrobial, etc.). These types of biological tests are crucial in the design and development of medical devices, specifically in assessing the biocompatibility of a material and its constituents. Overall, this knowledge gap provides an excellent opportunity to investigate the biological properties of Ti- and F-containing glasses and glass ceramics in a manner consistent with enhancing our ability to understand the individual and interaction effects between elements in order to predict material properties.

Although the primary inclusion criterion for this review was the incorporation of both Ti and F in the glasses/glass ceramics under investigation, the overwhelming majority of articles in the literature characterized the effects of only one of these constituents, primarily TiO_2_, on material properties. Specifically, just two glass articles reported findings on the effect of both Ti and F addition on one material, and these articles were published by the same author [58,59]. There were no glass-ceramic articles that investigated the effects of both Ti and F content within the same system. This is a result of employing trial and error and/or one variable at a time (OVAT) approaches to examining the influence of Ti and/or F substitutions in glass networks. These non-systematic approaches, whilst valuable to the literature, overlook the complexity of multi-component glasses and the likelihood for elements to interact with each other, forming synergistic relationships within the network that may influence material and host responses. These approaches complicate our ability to determine the individual and interaction effects of multiple glass constituents (e.g., Ti and F), especially in systems comprising numerous cations and anions with variable valences and cationic field strengths. As a result of the current literature teachings, it is extremely difficult to anticipate and understand the composition–structure–property relationships that arise from the inclusion of Ti and F (both individually and interactively) across broad compositional ranges. This, in turn, leads to long lead times in the development and commercial use of these materials [78]. Thus, based on the wide breadth of applicability for Ti-and F-containing glasses and glass ceramics, a more comprehensive understanding of these materials would be of extreme value in future materials design and development. As such, an approach that can simultaneously discover the effects of individual factors on material and host responses, as well as the synergistic relationships between interacting constituents, is required.

In this regard, the Materials Genome Initiative (MGI) has advanced a new paradigm for accelerated materials discovery [78]. Specifically, by combining experiment, theory, and computation in a systematic, high-throughput manner, we can strengthen our ability to support the a priori prediction of new materials with desirable physical, chemical, mechanical, and biological characteristics. One approach that is capable of characterizing these materials in a manner commensurate with the MGI is the Design of Mixtures (DoM) statistical modelling approach. The DoM approach has been employed across many industries to allow for unambiguous, systematic evaluations of the individual and interaction effects associated with various mixture components. This approach allows for the development of polynomial equations that indicate the relative influences of components on a given response and ultimately support the optimization of materials to a wide variety of properties via response surface regression methodologies [79,80,81,82,83]. These optimization studies can be used to correlate theoretical predictions to actual experimental results using minimal time and resources. This form of predictive modelling stands in contrast to traditional trial-and-error style approaches for materials discovery and has the potential to accelerate the design of glass and glass-ceramic materials via the simultaneous use of experimental methods and advanced modelling [78]. Specifically, the DoM approach can be employed to produce quantitatively predictive models relating to the composition–structure–property relationships in Ti- and F-containing glasses and glass ceramics with distinct compositions and variable glass-forming systems. By employing a simulation, modelling, and machine learning approach, these theoretical predictions can contribute to diverse digital datasets that can be accessible to researchers across the globe, thus encouraging collaboration and enhanced learning. As such, this program will enable the prediction of preferred material chemistries for a wide range of applications spanning nuclear, optical, electrochemical, dental, and industrial fields. This provides an exciting opportunity to contribute new knowledge to the broad scientific community surrounding the composition–structure–property relationships of glasses and glass ceramics modified with Ti and F.

## 6. Conclusions

There exist several ambiguities in the literature with respect to the effects that both Ti and F have on the properties of glasses and glass ceramics (individually and interactively). Specifically, the current literature lacks consistency, repeatability, and complete data on a range of glass chemistries and broad compositional ranges of Ti and F. Additionally, there is a discernible lack of information available as it pertains to the characterization of Ti- and F-containing glasses and glass ceramics for use in biomedical indications. Regrettably, the foundation for our existing knowledge of these materials has been based on the use of traditional non-systematic approaches. These approaches confound our understanding, lead to conflicting literature, and complicate our ability to determine the individual and interaction effects of multiple glass constituents. As a result, we are currently unable to accurately predict the effects of Ti and F on the physical, chemical, mechanical, and biological properties of glasses and glass ceramics with varying glass chemistries. This precludes the streamlined discovery, development, and deployment of these materials. To provide a more comprehensive understanding of glass and glass-ceramic systems modified with Ti and F and to support the accelerated design of these materials for use in a variety of indications, a systematic experimental approach, such as the Design of Mixtures approach, is strongly recommended.

## Figures and Tables

**Table 1:** Standard search parameters for PubMed and Web of Science.

Search #	Search String
1	Glass AND titanium AND fluoride
2	Glass AND titanium AND fluorine
3	Glass AND titanium AND fluorine NOT nano NOT film
4	Glass AND Ti AND F
5	Glass AND Ti AND fluoride
6	Glass AND TiO_2_ AND CaF_2_
7	Glass AND titanium tetrafluoride
8	Bioactive AND glass AND titanium AND fluoride

**Table 2:** Search results based on searches in Table 1.

Search String	Web of Science		Pubmed	
	Initial Returned Results	Articles Meeting Inclusion Criteria	Initial Returned Results	Articles Meeting Inclusion Criteria
1	127	5	34	0
2	423	1	71	0
3	96	2	47	0
4	2090	8	53	0
5	118	2	13	0
6	98	13	6	1
7	15	0	7	0
8	14	0	5	0
Total:	2981	31	236	1
Final Total:	3217 → 26 (after removing replicate articles across search strings)			

**Table 3:** Summary information on glass articles meeting inclusion criteria.

Verified Glasses								
Category		Source Title	Area of Application	Primary Experimental Methodologies	Composition(s) of Glass(es) Under Investigation	Melting Process	Reported Range of Ti	Reported Range of F
Single Primary Glass Former	B	Structural description of the Na_2_B_4_O_7_-Na_3_AlF_6_-TiO_2_ system. 1. IR and Raman study of the solidified melts (2007) [65]	Industrial (e.g., high-purity TiB_2_ powders)	XRD, FTIR, UV-Vis, Raman	Na_2_B_4_O_7_: Na_3_AlF_6_:TiO_2_	Melt quench. No further processing info available	0–11 wt.%/0–24.51 mol% TiO_2_	0–100 wt.%/0–75.49 mol% Na_3_AlF_6_
		Structural description of the Na_2_B_4_O_7_-Na_3_AlF_6_-TiO_2_ system. 2. A multinuclear NMR approach of melts and solids (2007) [66]	Industrial (same as above)	^11^B, ^27^Al, ^23^Na, ^19^F MAS NMR (solid and molten state)	Na_2_B_4_O_7_: Na_3_AlF_6_:TiO_2_	Melt quench. No further processing info available	0–11 wt.%/0–24.51 mol% TiO_2_	0–100 wt.%/0–75.49 mol% Na_3_AlF_6_
		Thermoluminescence, structural and magnetic properties of a Li_2_O-B_2_O_3_-Al_2_O_3_ glass system doped with LiF and TiO_2_ (2011) [17]	Nuclear (e.g., TL dosimetry)	XRD, AFM, thermoluminescence, EPR, DTA	LBA: 50Li_2_O·45B_2_O_3_·5Al_2_O_3_ LBA·0.225TiO_2_ LBA·50LiF, LBA·50LiF_2_·xTiO_2_ (wt.%)	Melt quench—melted at 1273 K for 10 min in platinum crucibles	0–0.325 wt.% TiO_2_	50 wt.% LiF_2_
		Influence of TiO_2_ ions on Spectroscopic Properties of Oxyfluoride Glasses (2019) [14]	Optics (e.g., photonics, scintillators)	XRD, Archimedes principle (density), UV-Vis, EPR, FTIR,	55B_2_O_3_·(25 − x)ZnF_2_·10CaF_2_·10Al_2_O_3_:xTiO_2_ (mol%)	Melt quench—melted at 1200 °C for 10 min in silica crucibles and annealed at 300 °C	0–0.9 mol% TiO_2_	10 mol% CaF_2_ and 24.1–25 mol% ZnF_2_
	F	IR spectroscopic study of the structure of glasses based on titanium oxyfluoride (2004) [67]	Materials Characterization	XRD, FTIR	40TiOF_2_·45BaF_2_·15MnF_2_ 40TiOF_2_·40BaF_2_·20MnF_2_ 30TiOF_2_·40BaF_2_·30MnF_2_ (mol%)	Melt quench—melted at 750–800 °C for 1 h	30–40 mol% of TiOF_2_	N/A (Primary glass former)
	P	FTIR spectra and thermal properties of TiO2-doped iron phosphate glasses (2015) [18]	Nuclear (e.g., immobilization of radioactive waste)	XRD, FTIR, DTA, Archimedes principle (density)	xTiO_2_·(90 − x)(60P_2_O_5_-40Fe_2_O_3_)·10CaF_2_ (mol%)	Melt quench—melted at 1200 °C for 2–3 h in porcelain crucible and annealed at 475 °C for 2 h	0–25 mol% TiO_2_	10 mol% CaF_2_
Mixed Primary Glass Formers	B/Si	Spectroscopic and Attenuation Shielding Studies on B_2_O_3_-SiO_2_-LiF-ZnO-TiO_2_ Glasses (2021) [19]	Nuclear (e.g., nuclear shielding)	XRD, UV-Vis, computation of nuclear parameters (e.g., polarizability, mean-free path, electron density), calculation of physical parameters (e.g., coordination number, glass transition temperature)	59B_2_O_3_·29SiO_2_-2LiF·(10 − x)ZnO xTiO_2_ (mol%)	Melt quench—held at 650 °C for 1 h then melted at 1200 °C and annealed at 450 °C for 2 h	0–10 mol% TiO_2_	2 mol% LiF
	B/P	Influence of TiO_2_ on structural, luminescent and conductivity investigations of CaF_2_-CaO-Y_2_O_3_-B_2_O_3_-P_2_O_5_ glasses (2019) [15]	Materials Characterization	XRD, Archimedes principle (density), calculation of physical properties (e.g., ionic radius, molar volume, refractive index), DTA, SEM-EDS, FTIR, Raman, EPR, UV-Vis, photoluminescence spectroscopy, measurement of DC ionic conductivity	(20-x)CaF_2_·10CaO·5Y_2_O_3_·10B_2_O_3_·55P_2_O_5_: xTiO_2_ (mol%)	Melt quench—melted at 1190–1215 °C for 1 h	0–1 mol% TiO_2_	19–20 mol% CaF_2_
	B/P	Thermoluminescence, elastic and dielectric investigations of calcium fluoro borophosphate glass composite materials doped by small concentrations of TiO_2_ (2021) [16]	Materials Characterization	XRD, density (method not specified), measurement of ultrasonic velocity, calculation of physical, elastic and mechanical properties (e.g., molar volume, field strength, Young’s, shear, bulk modulus, Poisson’s ratio, hardness), measurement of thermoluminescence emission, measurement of dielectric properties (e.g., dielectric constant and loss, AC conductivity, activation energy, density of energy states)	10B_2_O_3_·60P_2_O_5_·(30 − x)CaF_2_ xTiO_2_ (mol%)	Melt quench—melted at 1200–1225 °C for 40 min and annealed at 300 °C	0–1 mol% TiO_2_	29–30 mol% CaF_2_

XRD (X-ray powder diffraction), FTIR (Fourier transform infrared spectroscopy), UV-Vis (ultraviolet-visible spectroscopy), AFM (atomic force microscopy), EPR (electron paramagnetic resonance), DTA (differential thermal analysis), DSC (differential scanning calorimetry), MAS NMR (magic angle spinning nuclear magnetic resonance, SEM (scanning electron microscopy, Raman (laser Raman spectroscopy).

**Table 4:** Key experimental findings on the effects of Ti and F on the properties of glasses summarized from articles meeting inclusion criteria.

Verified Glasses			
Category		Source Title	Summary of Experimental Findings
Single Primary Glass Former	B	Structural description of the Na_2_B_4_O_7_-Na_3_AlF_6_-TiO_2_ system. 1. IR and Raman study of the solidified melts (2007) [65]	**XRD**: X-ray diffraction (XRD) analysis was conducted on several mixtures to determine the presence of crystalline species. The XRD patterns showed that a crystalline Na_3_AlF_6_ phase was present, in addition to an amorphous network, for all specimens. At a high TiO_2_ content (sample 0T11, 11 wt.% TiO_2_), the presence of rutile TiO_2_ was detected. At low TiO_2_ content (sample 80T11, 2.2 wt.% TiO_2_), no crystalline species were detected via XRD. The absence of brookite in all specimens was noted and ascribed to observations, which indicate that F^−^ ions can suppress its formation in TiO_2_ powders doped with fluorides. The presence of Anatase could not be confirmed due to peak overlap. **FTIR**: Three distinct regions in the vibrational spectra of borate glasses were identified, corresponding to (i) BO_3_ stretching (1500–1200 cm^−1^), (ii) BO_4_ stretching (1200–800 cm^−1^), and (iii) B-O-B bending (800–600 cm^−1^). The vibrational spectra of the 4- and 6-fold coordinated Al and Ti complexes were found to lie below 800 cm^−1^. The intensity of the band related to B_4_ units (1200–800cm^−1^) was found to directionally decrease, while the intensity of the region assigned to B3 units (1500–1200cm^−1^) increased, based upon increasing the TiO_2_ (which was similarly associated with an increase in fluorine). It is proposed that the formation of B4 groups in glasses with low TiO_2_ content (60–90% Na_2_B_4_O_7_) was due to the presence of TiO_4_ units, whereas the formation of B3 groups in glasses with high TiO_2_ content (20–50% Na_2_B_4_O_7_) was due to the presence of TiO_6_^2−^ units. The authors also suggested that the fluorine in these glasses played a role in changing the coordination of boron by substituting the oxygen around tetrahedral borate groups, leading to the formation of trigonal borate units and non-bridging oxygens. **Raman**: The study found that the band assigned to the Ti-O stretching mode of tetrahedral titanium units increased in intensity upon increasing the TiO_2_ content in the glass. This shift was observed to be 10cm^−1^ lower compared to a similar glass system, indicating the substitution of oxygen by fluorine around the tetrahedral Ti. Furthermore, the Raman band assigned to single Na_2_B_4_O_7_ shifted towards a lower frequency with increasing F content, suggesting that the substitution of oxygen by fluorine weakened the borate network by creating non-bridging oxygens. This weakening of the borate network was supported by an increase in the band assigned to B-O bonds attached to large borate rings (located between 1430–1490 cm^−1^) upon increasing the Ti and F content. The increase in non-bridging oxygens indicated a decreased network connectivity, suggesting that the addition of glass modifiers, such as Ti^4+^, disrupted large ring borate groups. **UV-Vis**: For readers interested in optical properties, please refer to the associated reference [65].
		Structural description of the Na_2_B_4_O_7_-Na_3_AlF_6_-TiO_2_ system. 2. A multinuclear NMR approach of melts and solids (2007) [66]	**^11^B MAS NMR**: The authors observed two prominent peaks in the NMR spectra, located at 0 and 15 ppm, which were associated with B4 and B3 sites, respectively. The proportion of tetrahedrally coordinated boron (B4) decreased as the TiO_2_ content increased and the Na_2_B_4_O_7_ content decreased, indicating a conversion from B4 to B3 units in regions enriched with TiO_2_. This conversion was proposed to occur through a coordination change in Ti, in which TiO_4/2_ + 2B(O,F)_4_ → Ti(O,F)_6_ + 2BO_3_. In samples with low TiO_2_ content (e.g., 80T11), the lack of high field strength Ti^4+^ ions for fluorine uptake led to increased uptake of fluorine by boron, resulting in the substitution of O^2−^ ions with F^−^ ions and the formation of non-bridging oxygens. This process caused the conversion of B4 units to B3 units. {19F}^11^B Rotational-Echo Double-Resonance (REDOR) experiments revealed the presence of oxyfluoro-species, mainly BO_2_F_2_, BO_3_F, and BO_2_F, in samples with low Na_2_B_4_O_7_ content (less than 50% Na_2_B_4_O_7_ and high TiO_2_), and BOF_2_ in samples with high Na_2_B_4_O_7_ content (more than 50% Na_2_B_4_O_7_ and low TiO_2_). **^19^F MAS NMR**: Valuable insights into fluorine’s preferential bonding behaviour, particularly concerning boron (B) and aluminium (Al) species, were examined. The study reveals fluorine’s distinct preference for higher field strength cations, resulting in a preference for F-B bonding over F-Al bonding, and the formation of F-B4 bonds instead of F-B3. Additionally, the investigation highlighted the interaction between fluorine and titanium (Ti) species in these systems, suggesting a compelling bonding preference of titanium for oxygen (O) rather than fluorine. The presence of oxyfluorotitanium species in the materials remains uncertain, as no detectable signals corresponding to Al-F-Ti, B-F-Ti, or F-Ti species were observed. **^27^Al MAS NMR**: A sharp Gaussian-type line centered at 0 ppm was observed in the reported spectra, indicating the presence of fluorinated AlF_6_^3−^ environments. Remarkably, in all ternary samples, excluding sample 80T11, >80% of aluminium atoms were found to be included in the fluorinated AlF_6_^3−^ phase. Additionally, the authors note that with increasing Na_2_B_4_O_7_ content, the amount of Al4 units also increased. The signal corresponding to Al5 species in these glasses (i.e., 35.5 ppm) was lower than the 36–45 ppm range expected for alumino-borate glasses. This difference was attributed to the substitution of oxygen by fluorine and the presence of oxyfluoro-species Al-(O,F)_5_. In the high Na_2_B_4_O_7_ region with relatively low TiO_2_ content, Ti^4+^ cations were not observed to compete with boron and aluminium atoms for fluorine. **^23^Na MAS NMR**: The two spectral peaks at 1 ppm and −8.1 ppm were attributed to the cryolite crystal phase, which was found to be present in all samples except for 80T11. {^19^F}^23^Na REDOR experiments also evidenced the presence of oxyfluoro-species, mainly NaO_7_F and NaO_6_F_2_ in samples with low Na_2_B_4_O_7_ content (i.e., <50 wt.% Na_2_B_4_O_7_) and NaO_6_F and NaO_5_F in samples with high Na_2_B_4_O_7_ content (above 50 wt.% Na_2_B_4_O_7_). **^27^Al high-temperature NMR**: In the low Na_2_B_4_O_7_ region (below 50% Na_2_B_4_O_7_) the addition of TiO_2_ decreased the average coordination state of aluminium atoms. Additionally, and while it is outside the scope of this review, the authors confirmed variations in high-temperature liquid structures and quenched structures. These variations were noted despite challenges and limited information from Lorentzian lineshape analysis. The authors indicate the value of in situ experiments as essential for understanding complex melt structures.
		Thermoluminescence, structural and magnetic properties of a Li_2_O-B_2_O_3_-Al_2_O_3_ glass system doped with LiF and TiO_2_ (2011) [17]	**XRD and AFM**: All specimens containing LiF exhibit the presence of LiF crystalline phases. Contrastingly, glasses *with* TiO_2_ and without LiF did not exhibit crystallinity per the methodology. A phase shift to smaller angles with the addition of 0.225wt.% TiO_2_ to the glass matrix doped with LiF was reported by the authors. The data indicate that the addition of TiO_2_ to the glass matrix doped with LiF causes an enlargement of the interplanar distance, ascribed to incorporation of Ti^4+^ ions in the LiF crystals within the 50Li_2_O-45B_2_O_3_-5Al_2_O_3_ (LBA) glass matrix. Ti^4+^ ions (68 pm) replace Li^+^ ions (60 pm) in LiF crystals, causing the observed changes in interplanar distance and crystal structure. The average diameter for the LiF crystalline particles found in the LBA + 50LiF + 0.225TiO_2_ sample were estimated to be 42.9nm. **DTA**: DTA curves were provided for samples LBA and LBA + 50LiF + 0.225TiO_2_. The glass transition (T_g_), onset of crystallization (T_x_), and peak of crystallization (T_c_) temperatures for sample LBA were around 650K, 717K, and 738K. Upon the addition of LiF and TiO_2_, these temperatures decreased to 616K, 667K, and 702K, respectively. There was no further evidence on whether this change in thermal properties arose from the effects of LiF, TiO_2_ or both constituents on network structure. **EPR**: EPR spectra were obtained for non-irradiated and 5 kGy ^60^Co γ-ray irradiated samples. No signs of EPR were detected for the non-irradiated sample (LBA + 50LiF + 0.225TiO_2_). Conversely, irradiated LBA and LBA + 50LiF samples exhibited four equidistant signals at approximately 12.5 + 0.2 mT with a g value of 2.0149. With increasing TiO_2_ concentrations, a signal emerged at g = 2.0098, attributed to Ti^3+^ center formation due to ^60^Co γ-ray radiation. This signal peaked at 0.225 wt.% TiO_2_ and decreased thereafter. **Thermoluminescence**: For readers interested in nuclear and/or thermoluminescent properties, please refer to the appropriate reference [17].
		Influence of TiO_2_ ions on Spectroscopic Properties of Oxyfluoride Glasses (2019) [14]	**XRD**: The XRD patterns for both undoped and TiO_2_-doped glasses were representative of fully amorphous materials with no detectable crystalline species. **Density**: The densities of the glasses varied from 2.8678 and 2.9162 g/cc. Reductions in density were associated with increasing TiO_2_ concentration. Titanium oxide gradually replaced zinc fluoride in the glass composition, and since titanium oxide has a lower density and lighter atomic weight compared to zinc fluoride, the overall density of the glass decreased as more TiO_2_ was added. **Physical Properties**: Various physical parameters were computed from the measured density. As the concentration of TiO_2_ increased, the molar volume of samples increased (82.14–81.93 g/mol) in accordance with the decreased density. From this trend in molar volume, it is expected that the Ti ions promoted the formation of non-bridging oxygens, thus disrupting the network and causing a loosely packed structure. Furthermore, with increasing TiO_2_ content, there was a decrease in interatomic distance (36.2–17.67 ^0^A), which suggested that the addition of TiO_2_ to the glass network caused the atoms to become more tightly packed. There was an increase in field strength (1.0061–4.4601 × 10^15^ cm^−2^) upon the increasing content of TiO_2_. Additional physical properties established in the literature included molecular weight, titanium concentration, polaron radius, and optical basicity. For readers interested in this additional information, refer to [14]. **FTIR**: In the B_2_O_3_-ZnF_2_-CaF_2_-Al_2_O_3_ glass network, four distinct bands are observed, each originating from specific borate groups: ~1350 cm^–1^ for B-O stretching in BO_3_ units within pyroborate units, ~1220 cm^–1^ for B-O stretching in BO_3_ units within orthoborate units, ~1010 cm^–1^ for bending of BO_4_ units, and ~685 cm^–1^ for B-O-B stretching. When TiO_2_ is added to the glass composition, two additional bands emerge, attributed to titanium structural groups: ~770 cm^–1^ for B-O-Ti or TiO_4_ and ~650 cm^–1^ for Ti-O-Ti or TiO_6_. As the concentration of TiO_2_ increases up to 0.7 mol% in the glass, the intensity of the band associated with TiO_6_ (octahedral) gradually grows at the expense of the TiO_4_ tetrahedral band. Likewise, with an increasing concentration of TiO_2_, the intensity of the band related to BO_3_ structural units increases while the band related to BO_4_ structural units diminishes in intensity. The overall findings suggest that titanium acts as a network modifier in these glasses. The results also indicate that titanium ions within the glasses exist in two states: Ti^4+^ (found in both tetrahedral and octahedral positions) and Ti^3+^ (located in octahedral sites). **EPR**: The EPR spectra of all samples containing TiO_2_ showed signal at approximately g = 1.930. As the concentration of TiO_2_ increased (up to 0.7 mol%), the half-width and intensity of this signal increased. This signal was attributed to the 3d1 unpaired electron of paramagnetic Ti^3+^ ions, thus suggesting that up to 0.7 mol% TiO_2_, there is an increase in Ti^3+^ ions in these glasses. **UV-Vis**: For readers interested in optical properties, refer to [14].
	F	IR spectroscopic study of the structure of glasses based on titanium oxyfluoride (2004) [67]	**XRD**: Glasses were reported as being free from identifiable crystalline species. **FTIR**: FTIR analysis was performed to gain insight on the main structural groups of the TiOF_2_·BaF_2_·MnF_2_ glasses. Results revealed intense bands in the ranges 700–900 cm^–1^ (stretching of Ti-O-Ti and Ti=O), 600–500 cm^–1^ (Ti-F stretching), 350–200 cm^–1^, and a weaker band at 130 cm^–1^. It was shown that the structural networks of the glasses were composed of TiOF_5_ and TiO_2_F_4_ polyhedral joined by fluoride and oxygen bridges. Three intense bands were shown on FTIR spectra in the ranges 700–900 cm^–1^ (stretching of Ti-O-Ti and Ti=O), 600–500 cm^–1^ (Ti-F stretching), 350–200 cm^–1^ (superposition of peaks, believed to arise from the bending vibrations of F-Ti-F bonds, F-Ti-O, Ti-O-Ti, Mn-F and/or Mn-F-Ti bonds). These results suggested that the structure of the glasses under investigation were composed of TiOF_5_ and TiO_2_F_4_ polyhedral joined by fluoride and oxygen bridges. Based on the FTIR spectra, the authors suggested that in compositions with higher content TiO_2_, the presence of TiOF_5_ polyhedra was greater than TiO_2_F_4_ polyhedra.
	P	FTIR spectra and thermal properties of TiO_2_-doped iron phosphate glasses (2015) [18]	**XRD**: Results show no crystalline phases within glasses with increasing TiO_2_ content up to 25 mol%. **FTIR**: The appearance of a new FTIR peak at approximately 651 cm^−1^ upon adding TiO_2_ to the base glass composition confirmed the presence of stretching modes of Ti-O in octahedral titanium units. The intensity of this band increased with higher concentrations of TiO_2_ in the glasses. Additionally, as the TiO_2_ content increases, the bands associated with various P=O bonds decrease in area and shift to higher energy. These results indicate that titanium ions modify the phosphate network by breaking P=O bonds and forming P-O-Ti bonds, leading to increased cross-linking, and strengthening of the phosphate network. **DTA**: T_g_ increases (from 546 to 612 °C) as the TiO_2_ content in the glasses is increased. Several factors contribute to this increase in T_g_. Firstly, the replacement of P=O bonds by Ti-O-P bonds plays a role. Secondly, the titanium ions act as cross-linkers, connecting the phosphate chains more effectively, which enhances the structural connectivity of the glasses and thus raises the T_g_. The difference between the glass transition temperature (T_g_) and the onset of crystallization peak (T_r_), denoted as (T_r_-T_g_), decreases with an increase in TiO_2_ content. This suggests an increased tendency for crystallization in the Ti-containing glasses. The stronger cation field strength of Ti^4+^ is responsible for this effect, as it causes atoms to arrange periodically over longer distances, promoting longer-range order and crystallization. **Density**: The density increases from 3.16 to 3.31 g/cm^3^ as the TiO_2_ content is increased. The role of titanium in the glass structure is classified as an intermediate oxide according to Dietzel’s classification. Based on this classification, the influence of TiO_2_ on density can be explained in two ways: Firstly, TiO_2_ may act as a network former and thereby contribute to building the basic framework of the glass structure. On the other hand, TiO_2_ may act as a network modifier, in which Ti^4+^ ions act as ionic cross-links between phosphate units, strengthening the glass network. In this case, titanium would enhance the connectivity between the phosphate units and modify the existing glass structure. The increase in TiO_2_ leads to a higher cross-linking density between phosphate units, which, in turn, results in an increase in the overall bulk density of the glasses.
Mixed Primary Glass Formers	B/Si	Spectroscopic and Attenuation Shielding Studies on B_2_O_3_-SiO_2_-LiF- ZnO-TiO_2_ Glasses (2021) [19]	**XRD**: XRD verified the amorphous state of the glasses and the absence of identifiable crystalline species. **Physical Properties**: The average silica coordination number and bulk modulus increased as ZnO was gradually substituted by TiO_2_. With increasing TiO_2_ (and decreasing ZnO), the molar volume, molar refractivity, inter-ionic distance, inter-nuclear distance, polaron radius, Ti–Ti separation decreased. T_g_ increased with increasing TiO_2_ content (673–685 K). Additional physical properties established in the literature included cohesive energy, ionic concentration, theoretical bandgap, ionicity, mechanical constraints, floppy modes, lone-pair electrons, metallization criterion, number of bonds, electronegativity, covalency, molar refractivity, molar polarizability, reflection loss, and electron polarizability. For readers interested in this additional information, refer to [19]. **Density**: The number of bonds per unit area and cross-linking density increased with increasing TiO_2_ content (8.52–11.4 × 10^29^ m^−3^ and 2.11–2.42, respectively). **UV-Vis and Nuclear Parameters**: For readers interested in optical and nuclear shielding properties, please refer to the appropriate reference [19].
	B/P	Influence of TiO_2_ on structural, luminescent and conductivity investigations of CaF_2_-CaO-Y_2_O_3_-B_2_O_3_-P_2_O_5_ glasses (2019) [15]	**XRD**: XRD verified the amorphous nature of all glass samples and their absence from any identifiable crystalline species. This observation was further reinforced using EDS and SEM. **Physical Properties**: With increasing TiO_2_, the molar volume, ion concentration, and field strength increased. Additional physical properties established in the literature included ionic concentration, ionic radius, polaron radius, electronegativity, optical basicity, polarizability, and refractive index. For readers interested in this additional information, refer to [15]. **Density**: The density values decreased (2.893–2.8055 g/cm^3^) with increasing TiO_2_ content (up to 1 mol%). **DTA**: Upon increasing the TiO_2_ content up to 0.6 mol%, the T_g_ values decreased from 445 to 440 °C, whereafter they increased slightly back to 442 °C. Similarly, the crystallization temperature (T_c_) of the exothermic peak decreased from 894 to 886 °C upon TiO_2_ addition up to 0.6 mol%, whereafter it increased slightly back to 889 °C. As a result, T_c_–T_g_ decreased upon increasing TiO_2_ wt.% composition up to 0.6 mol%, where afterwards, the T_c_–T_g_ values were increased with respect to the TiO_2_ content. **FTIR**: When TiO_2_ is added to the glass samples (up to 0.6 mol%), the intensity of peaks corresponding to various bands of borate and phosphate units (e.g., P-O-P asymmetrical units, PO_2_^−^ asymmetrical units, BO4 units, B-O-B linkages, BO3 units) increases. However, beyond this TiO_2_ concentration, these intensities start to decrease. In contrast, the intensity of the peak attributed to P-O-P symmetrical units decreases as the TiO_2_ content is increased up to 0.6 mol% whereafter for TiO_2_ content beyond 0.6 mol% (0.8 and 1 mol%), this intensity increases. Additionally, as the TiO_2_ content increases, the band attributed to TiO_4_ structural units shifts to higher wavenumbers, while the band attributed to TiO_6_ structural units shifts to lower wavenumbers. **Raman**: The raw data from Raman analyses showed inconsistencies with the discussion presented by Rao and Kumar. For example, the authors stated that the observed different phosphate structural units such as PO_3_^2−^/P-O-P asymmetric and P-O-P symmetrical units were observed around 1009–1040 and 674–686 cm^–1^. However, based on the Raman spectra, the PO_3_^2−^, P-O-P symmetrical, and the P-O-P asymmetrical bands are located at around 925–940cm^−1^, 600–625 cm^−1^, and 960–990 cm^−1^, respectively. Based on this ambiguity, the Raman data were not considered further in this review. **EPR**: The EPR spectra showed a strong signal at a g value of 1.96, which has been attributed to the Ti^3+^ ions in octahedral configuration. The integrated area under the EPR curve was plotted against TiO_2_ content in the glasses and indicated the signal was greatest at 0.6 mol% TiO_2_. This suggested that the exchange from Ti^4+^ ions to Ti^3+^ was greatest up to 0.6 mol% TiO_2_, whereafter the opposite exchange took place. **UV-Vis, Photoluminescence Spectroscopy, DC conductivity**: For readers interested in optical and/or electric properties, please refer to the appropriate reference [15].
	B/P	Thermoluminescence, elastic and dielectric investigations of calcium fluoroborophosphate glass composite materials doped by small concentrations of TiO_2_ (2021) [16]	**XRD**: XRD patterns confirm the amorphous nature of all glass compositions and the absence of identifiable crystalline species. **Density**: Density values increased (2.812–2.831 g/cm^3^) with increasing TiO_2_ content (up to 1 mol%). **Physical Properties**: The molar volume, and field strength increased with increasing TiO_2_ content. Contrastingly, the inter-ionic distance and polaron radius decreased with increasing TiO_2_ concentration. **Elastic and Mechanical Properties**: Young’s modulus, shear modulus, bulk modulus, Poisson ratio, and microhardness values were calculated using ultrasonic velocity measurements. Young’s modulus, shear modulus, bulk modulus, and microhardness values increased with increasing TiO_2_ concentration up to 0.6 mol%, whereas the Poisson ratio values decreased. The increase in these parameters upon TiO_2_ addition up to 0.6 mol% was believed to be due to the substitution of divalent Ca^2+^ ions with tetravalent Ti^4+^ ions as well as the substitutions of P-O-P and B-O-B bonds for stronger P-O-Ti and/or B-O-Ti bonds. **Thermoluminescence, Dielectric Properties**: For readers interested in thermoluminescent and/or dielectric properties, please refer to the appropriate reference [16].

**Table 5:** Summary information on glass-ceramic articles meeting inclusion criteria.

Verified Glass Ceramics								
Category		Source Title	Area of Application	Primary Experimental Methodologies	Composition(s) of Glass Ceramic(s) Under Investigation	Melting Process (Before Heat Treatment)	Reported Range of Ti	Reported Range of F
Single Primary Glass Former	P	X-Ray-Powder Diffraction of Crystalline Phases in Phosphate Bioglass Ceramics (1994) [28]	Materials Characterization	XRD, TEM, EDX, NMR	(11–18)Na_2_O·(13–19)CaO·(6–18)Al_2_O_3_·(45–55)P_2_O_5_·(1–2)F with (2–5)TiO_2_ and/or (1.5–5)ZrO_2_ and/or (3–6)FeO/Fe_2_O_3_ (wt.%) *	Melt quench. No further processing info available	2–5 wt.% TiO_2_	1–2 wt.% F^−^
	Si	The effect of additives on the crystallization of Na_2_O-CaO-MgO-Al_2_O_3_-SiO_2_-TiO_2_ system glasses (1999) [29]	Materials Characterization	XRD, DTA, SEM	Sample 1: 12Na_2_CO_3_·6CaCO_3_·6MgO·12Al_2_O3·52SiO_2_·6TiO_2_ (wt.%) Sample 2: Sample 1·6CaF_2_ (mol%) Sample 3: Sample 1·6Na_2_CO_3_ (mol%) Sample 4: Sample 1·6CaF_2_·5B_2_O_3_ (mol%)	Melt quench—melted in alumina crucible at 1450–1500 °C for 2 h and annealed at 600 °C	6 wt.% TiO_2_	6 mol% CaF_2_
	P	Preparation of apatite-containing calcium phosphate glass-ceramics (2007) [32]	Dental (e.g., dental fillers)	XRD, DTA, SEM, Raman	CTP-F: 40CaO·25TiO_2_·30P_2_O_5_·5CaF_2_ (mol%) CTP: 45CaO·25TiO_2_·30P_2_O_5_ (mol%)	Melt quench—melted in a platinum crucible at 1300 °C for 0.5 h	25 mol% TiO_2_	5 mol% CaF_2_
	P	Induced crystallization and physical properties of Li_2_O-CaF_2_-P_2_O_5_: TiO_2_ glass system—Part I. Characterization, spectroscopic and elastic properties (2008) [25]	Materials Characterization	XRD, SEM, DTA, Archimedes principle (density), evaluation of weight loss in water (chemical durability), FTIR, Raman, measurement of ultrasonic velocity, calculation of elastic and mechanical properties (e.g., Young’s and shear modulus, Poisson’s ratio, hardness)	(30 − x)Li_2_O·10CaF_2_·60P_2_O_5_·xTiO_2_ (mol%)	Melt quench—melted in platinum crucible at 1000 °C for 2 h and annealed at 250 °C	0–0.8 mol% TiO_2_	10 mol% CaF_2_
	P	Induced crystallization and physical properties of Li_2_O-CaF_2_-P_2_O_5_: TiO_2_ glass system—Part II. Electrical, magnetic and optical properties (2008) [68]	Materials Characterization	UV-Vis, EPR, measurement of dielectric properties (e.g., dielectric constant and loss), Guoy’s method (magnetic susceptibility)	(30 − x)Li_2_O·10CaF_2_·60P_2_O_5_:xTiO_2_ (mol%)	Melt quench—melted in platinum crucible at 1000 °C for 2 h and annealed at 250 °C	0–0.8 mol% TiO_2_	10 mol% CaF_2_
	P	Luminescence spectroscopy of Ti ions in Li_2_O-CaF_2_-P_2_O_5_ glass ceramics (2008) [69]	Optics (e.g., luminescence emission)	XRD, UV-Vis, fluorescence spectroscopy (luminescence emission)	(30 − x)Li_2_O·10CaF_2_·60P_2_O_5_·xTiO_2_ (mol%)	Melt quench—melted in platinum crucible at 1000 °C for 2 h and annealed at 250 °C	0–0.8 mol% TiO_2_	10 mol% CaF_2_
	P	Preparation of a Calcium Titanium Phosphate Glass-Ceramic with Improved Chemical Durability (2009) [33]	Dental (e.g., fissure sealant)	XRD, Raman, SEM-EDX, DTA, MAS-NMR, ICP-AES (ion release)	CTP-F: 35CaO·10CaF_2_·30P_2_O_5_·25TiO_2_ CTP: 45CaO·25TiO_2_·30P_2_O_5_ (mol%)	Melt quench—base glass (no CaF_2_) was heated in Pt crucible at 1000 °C for 30 min. CaF_2_ was added, then melted at 1300 °C for 20 min	25 mol% TiO_2_	10 mol% CaF_2_
	P	Fabrication and luminescence behaviour of phosphate glass ceramics co-doped with Er^3+^ and Yb^3+^ (2012) [70]	Optics (e.g., up conversion, near infrared emission)	XRD, UV-Vis, fluorescence spectroscopy (luminescence emission)	60P_2_O_5_·29.8Li_2_O·10CaF_2_·0.2TiO_2_·0.25Er_2_O_3_·5Yb_2_O_3_ (mol%)	Melt quench—melted in corundum crucible at 1300 °C for 1.5 h and annealed at 400 °C	0.2mol% TiO_2_	10mol% CaF_2_
	P	Synthesis of nanostructured Li_3_Me_2_(PO_4_)_2_F_3_ glass-ceramics (Me = V, Fe, Ti) (2016) [27]	Electrochemistry (e.g., nanostructured cathode materials for Na-ion batteries)	XRD, DTA, SEM, impedance spectroscopy (electrical conductivity)	VV: Li_3_V_2_(PO_4_)_2_F_3_ F_0_._5_V_0_._5_: Li_3_Fe_0_._5_V_1_._5_(PO_4_)_2_F_3_ FV: Li_3_FeV(PO_4_)_2_F_3_ FF: Li_3_Fe_2_(PO_4_)_2_F_3_ TT: Li_3_Ti_2_(PO_4_)_2_F_3_ TV: Li_3_TiV(PO_4_)_2_F_3_	Melt quench—glass was heated at 200 °C for 2 h (no LiF), LiF was added, then melted in alumina crucible, inside a larger graphite crucible with a lid at 1300 °C for 15 min	TT: Li_3_Ti_2_(PO_4_)_2_F_3_ TV: Li_3_TiV(PO_4_)_2_F_3_	
	P	Syntheses and nanocrystallization of NaF–M_2_O_3_–P_2_O_5_ NASICON-like phosphate glasses (M = V, Ti, Fe) [71]	Electrochemistry (same as above)	XRD, DTA, Scherrer formula (calculated grain size), FESEM, impedance spectroscopy (e.g., impedance, conductivity, activation energy), Hebb-Wagner method (electronic and ionic transference numbers)	VV: Na_3_V_2_(PO_4_)_2_F_3_ TT: Na_3_Ti_2_(PO_4_)_2_F_3_ FF: Na_3_Fe_2_(PO_4_)_2_F_3_ TV: Na_3_TiV(PO_4_)_2_F_3_ FV: Na_3_FeV(PO_4_)_2_F_3_ FT: Na_3_FeTi(PO_4_)_2_F_3_	Melt quench—glass was heated at 240 °C for 2 h (no NaF), NaF was added, then melted in alumina crucible, inside a larger graphite crucible with a lid, at 1300 °C for 15 min	TT: Na_3_Ti_2_(PO_4_)_2_F_3_ TV: Na_3_TiV(PO_4_)_2_F_3_ FT: Na_3_FeTi(PO_4_)_2_F_3_	
	Si	Influence of TiO_2_ content on phase evolution, microstructure and properties of fluorcanasite glass-ceramics prepared through sintering procedure for dental restoration applications (2018) [72]	Dental (e.g., ceramic restorations)	XRD, DTA, dilatometry, FESEM-EDX, Archimedes method (e.g., linear shrinkage, bulk density, sinterability), chemical solubility, micro indentation, three point bending method (flexural strength), Chantikul method (fracture toughness)	57.1SiO_2_·7.4 K_2_O·9.8 Na_2_O·13.3CaO·12.4CaF_2_·xTiO_2_ (wt.%)	Melt quench—melted in alumina crucibles at 1350 °C for 1 h	0, 6, 9, 12 wt. ratios TiO_2_	12.4 wt.% CaF_2_
	Si	Effect of complex nucleation agents on preparation and crystallization of CaO-MgO-Al_2_O_3_-SiO_2_ glass-ceramics for float process (2016) [26]	Materials Characterization	XRD, Raman, DSC, SEM, FESEM	T: 9CaO·6MgO·21Al_2_O_3_·49SiO_2_·4K_2_O·4Na_2_O·2ZnO_2_·5TiO_2_ TF: T·2CaF_2_ TP: T·2P_2_O_5_ TFP: T·2CaF_2_·2P_2_O_5_ (wt.%)	Melt quench—melted in platinum crucible at 1470–1520 °C for 3 h and annealed at 600 °C for 30 min	5 wt.% TiO_2_	0–2 wt.% CaF_2_
	Si	Crystallization and microstructure of CaO-MgO-Al_2_O_3_-SiO_2_ glass-ceramics containing complex nucleation agents (2014) [30]	Materials Characterization	XRD, DTA, SEM, chemical solubility	F: 14CaO·10MgO·12Al_2_O_3_·50SiO_2_·2K_2_O·3Na_2_O·1SbO_3_·8CaF_2_ FT: F·3TiO2 FZ: F·3ZrO_2_ FP: F·3P_2_O_5_ (wt.%)	Melt quench—melted at 1400 °C for 4 h and annealed at 550 °C for 1 h	3 wt.% TiO_2_	8 wt.% CaF_2_
Mixed Primary Glass Formers	Si/P	The effect of TiO_2_ concentration on properties of apatite-mullite glass-ceramics for dental use (2016) [73]	Dental (e.g., metal-free dental replacement materials)	XRD, DTA, SEM-EDX, XRF, chemical solubility, measurement of mechanical properties (flexural strength)	HGF1Ti0.0: 34.62SiO_2_·23.08Al_2_O_3_·11.54P_2_O_5_·23.08CaO·7.69CaF_2_·0TiO_2_ HGF1Ti0.5: 34.43SiO_2_·23.95Al_2_O_3_·11.48P_2_O_5_·22.95CaO·7.65CaF_2_·0.53TiO_2_ HGF1Ti1: 34.25SiO_2_·22.83Al_2_O_3_·11.42P_2_O_5_·22.83CaO·7.61CaF_2_·1.07TiO_2_ HGF1Ti1.5: 34.09SiO_2_·22.73Al_2_O_3_·11.36P_2_O_5_·22.73CaO·7.58CaF_2_·1.51TiO_2_ HGF1Ti2: 33.91SiO_2_·22.61Al_2_O_3_·11.30P_2_O_5_·22.61CaO·7.54CaF_2_·2.03TiO_2_ HGF1Ti2.5: 33.73SiO_2_·22.49Al_2_O_3_·11.24P_2_O_5_·22.49CaO·7.50CaF_2_·2.51TiO_2_ HGF1Ti3: 33.58SiO_2_·22.39Al_2_O_3_·11.19P_2_O_5_·22.39CaO·7.46CaF_2_·2.98TiO_2_ HGF1Ti3.5: 33.41SiO_2_·22.27Al_2_O_3_·11.14P_2_O_5_22.27CaO·7.42CaF_2_·3.48TiO_2_ HGF1Ti4: 33.21SiO_2_·22.14Al_2_O_3_·11.07P_2_O_5_·22.14CaO·7.38CaF_2_·4.05TiO_2_ HGF1Ti4.5: 33.06SiO_2_·22.04Al_2_O_3_·11.02P_2_O_5_·22.04CaO·7.35CaF_2_·4.48TiO_2_ HGF1Ti5: 32.85SiO_2_·21.90Al_2_O_3_·10.95P_2_O_5_·22.90CaO·7.30CaF_2_·5.01TiO_2_ (wt.%)	Melt quench—melted in alumina crucible at 1050 °C for 1 h then 1450 °C for 2 h. Glass frit was remelted at 1450 °C for 2 h and annealed at 650 °C for 1 h	0–5.01 wt.% TiO_2_	7.3–7.69 wt.% CaF_2_
	Si/B	Influence of TiO_2_ content on the crystallization and microstructure of machinable glass-ceramics (2016) [31]	Materials Characterization	XRD, DSC, FTIR, SEM, measurement of mechanical properties (hardness)	MGT-1: 35.59SiO_2_·7.94Al_2_O_3_·14.96MgO·5.34K_2_O·4.33B_2_O_3_·31.75MgF_2_·0.62TiO_2_ MGT-2: 32.54SiO_2_·7.94Al_2_O_3_·14.96MgO·5.34K_2_O·4.33B_2_O_3_·31.75MgF_2_·3.15TiO_2_ MGT-3: 31.6SiO_2_·5.94Al_2_O_3_·14.96MgO·5.34K_2_O·4.33B_2_O_3_·31.75MgF_2_·6.27TiO_2_ (mol%)	Melt quench—melted in alumina crucible at 1500 °C for 2 h and annealed at 630 °C for 2 h	0.62, 3.15, 6.27 mol% (1, 5, 10 wt.%) TiO_2_	31.75 mol% (12 wt.%) MgF_2_
	Si/P	On the microstructure of biocomposites sintered from Ti, HA and bioactive glass [74]	Hard Tissue Regeneration	XRD, TEM-EDS	BG: 48SiO_2_-10Na_2_O-13CaO-9.2P_2_O_5_-9.7B_2_O_3_-4.87MgO-4.25TiO_2_-0.98CaF_2_ (wt.%)	Glass was heat treated at 800 °C for 2 h. No additional information available on synthesis of glass	4.25 wt.% TiO_2_	0.98 wt.%

* Specific compositions not provided. EPR (electron paramagnetic resonance), FESEM (field emission scanning electron microscopy), TEM (transmission electron microscopy, EDX (energy dispersive X-ray spectroscopy), ICP-AES (inductively coupled plasma atomic emission spectroscopy), XRF (X-ray fluorescence).

**Table 6:** Key experimental findings on the effects of Ti and F on the properties of glass ceramics summarized from articles meeting inclusion criteria.

**Verified Glass Ceramics**			
**Category**		**Source Title**	**Key Experimental Findings**
Single Primary Glass Former	P	X-Ray-Powder Diffraction of Crystalline Phases in Phosphate Bioglass Ceramics (1994) [28]	**XRD**: X-ray diffraction (XRD) patterns of the glass ceramic containing TiO_2_ and ZrO_2_ (labelled glass 1) revealed eight crystalline phases in the temperature range of 500–750 °C: NaZr_2_(PO_4_)^3^, AlPO_4_-tridymite, NaCaPO_4_, Ca_5_(PO_4_)^3^(F/OH) (apatite), and four new phosphate phases named U1, U2, U3, and U4. In the XRD patterns of the glass ceramic containing only ZrO_2_ (labelled glass 2), five crystalline phases were identified in this temperature range: AlPO_4_, Ca_5_(PO_4_)^3^(F/OH) (apatite), U2, U3, and AlPO_4_-berlinite. On the other hand, XRD patterns of the glass ceramics doped with ZrO_2_ and FeO/Fe_2_O_3_ (labelled glass 3) and only FeO/Fe_2_O_3_ (labelled glass 4) revealed seven and five crystalline phases, respectively, in the temperature range of 500–750 °C. The U2 and U3 phases were present in all glass-ceramic samples, including the base glass. Moreover, the phosphate phases (U phases) were exsolved at higher temperatures in glass ceramics containing ZrO_2_ and TiO_2_ compared to glass ceramics containing FeO/Fe_2_O_3_. **TEM-EDX**: Approximate compositions of the new phosphate crystalline phases were proposed: U1: Na_x_Ca_(1−x)_[(Zr, Ti)_(1+x)_Al_(1−x)_](PO_4_)_3_, U2: CaNa_3_Al(P_2_O_7_)_2_, U3: CaNa_5_Al_3_(P_2_O_7_)_4_, U4: CaNa_3_Al(P_2_O_7_)_2_. **^31^P-MAS NMR**: NMR revealed that the untreated glass contained approximately 10% disphosphate and 90% monophosphate phases, wherein U1 was a monophosphate and U2, U3, and U4 were disphosphates.
	Si	The effect of additives on the crystallization of Na_2_O-CaO-MgO-Al_2_O_3_-SiO_2_-TiO_2_ system glasses (1999) [29]	**XRD**: It was observed that the heat treatment of the base glass (sample no. 1) resulted in the formation of both fassaite and anorthite. The appearance of two exothermic peaks at 721 and 820 °C on DTA was attributed to the crystallization of these fassaite and anorthite phases, respectively. To investigate the effect of CaF_2_ doping on crystallization, the glasses were subjected to the same heat treatment. In sample no. 2 (doped with CaF_2_ only), this led to the exclusive formation of fassaite but in lesser amounts compared to the undoped base glass. The second method of heat treatment for sample no. 2 resulted in the formation of fluorite in addition to fassaite. Interestingly, the amount of fassaite formed was consistent with the previous sample, suggesting that the presence of fluorite did not significantly affect fassaite formation. The absence of anorthite in both heat treatment scenarios indicated that CaF_2_ hindered the nucleation and growth of anorthite, possibly due to the preferential formation of fluorite. When sample no. 4 (doped with CaF_2_ and B_2_O_3_) was subjected to the second heat treatment method, both fassaite and anorthite crystals were identified in similar amounts present in the base glass, and no fluorite was present. This suggested that B_2_O_3_ hindered the nucleation and growth of fluorite. **DTA**: DTA was performed on sample no. 1 (base glass) to determine the heat treatment method for the glasses. Two exothermic peaks (~721 and ~820 °C) suggested at least two main crystals were produced in the crystallization process. Based on the DTA analysis, two heat treatment methods were determined: (1) hold sample at 700 °C for 1 h for nucleation then hold at 1000 °C for 2 h for growth, and (2) hold sample at 800 °C for 1 h for nucleation then hold at 1000 °C for 2 h for growth. **SEM**: SEM analysis compared the crystalline structure of samples heat treated using the two methods. For sample no. 1 (base glass) heat treated with the first method, SEM photographs revealed the presence of some 0.25 µm crystal grains. In contrast, CaF_2_-containing samples (sample no. 2) heat treated using the same method showed smaller crystal grains compared to the base glass. When sample no. 2 was heat treated using the second method, dendritic crystal growth was observed, attributed to the formation of fluorite crystals. For sample no. 4 containing both B_2_O_3_ and CaF_2_, SEM photographs displayed more uniformly distributed crystal grains compared to the base glasses. Na_2_O and CaF_2_ have contrasting effects on the nucleation and growth of fassaite and anorthite crystals. Na_2_O does not significantly impact anorthite, while CaF_2_ suppresses the nucleation and growth of fassaite and instead promotes the formation of fluorite crystals. CaF_2_ addition introduces F^−^ ions into the glass network as [SiO_3_F] or [AlO_3_F] units, leading to a decrease in the number of [SiO_4_] units, which are basic structural units of fassaite and anorthite. Additionally, Na_2_O, a typical glass network modifier, induces phase separation when added to the base glasses. In contrast, B_2_O_3_ inhibits fluorite formation and enhances the nucleation and growth of both fassaite and anorthite in the presence of CaF_2_. When both CaF_2_ and B_2_O_3_ are added to the base glasses, F^−^ ions replace O^2−^ in the [BO_3_] unit, transforming it into [BO_2_F]. Due to an equal number of F^−^ ions and B^3+^ ions, all F^−^ ions are in the form of [BO_2_F]. The strong B-F bond energy hinders the formation of CaF_2_ crystals.
	P	Preparation of apatite-containing calcium phosphate glass-ceramics (2007) [32]	**XRD**: Analysis of both CTP (CaTi_4_(PO_4_)^6^) and CTP-F (CaTi_4_(PO_4_)^6^ with CaF_2_ addition) glass ceramics revealed the presence of peaks corresponding to NASICON-type crystal structure, which is attributed to CaTi_4_(PO_4_)^6^. Additionally, peaks assigned to Ti(PO_3_)_3_ and (TiO)_2_P_2_O_7_ crystals were also observed. In the CTP-F glass-ceramic, the crystalline phase composition was more diverse; along with NASICON-type and titanium phosphate crystals, apatite and TiO_2_ (anatase) crystals were also present. The introduction of CaF_2_ (CTP-F) influenced the crystallization behaviour, leading to the preferential formation of apatite crystals. However, this addition resulted in a decrease in the peak intensity of the CaTi_4_(PO_4_)^6^ crystal compared to that in the CTP glass-ceramic. **DTA**: Differential thermal analysis was performed to determine the heat treatment process for the glasses. The CTP-F glass was held at 700 °C for 24 h for nucleation and subsequently heated to 755 °C for 12 h for crystal growth. The CTP glass was held at 695 °C for 24 h for nucleation and 735 °C for 12 h for crystal growth. **SEM**: SEM micrographs revealed the surfaces of both CTP and CTP-F glass ceramics after chemical etching with 1 N HCl for 5 min at room temperature. The results indicated a stark contrast in the etching behaviour between the two materials. In the case of CTP-F glass-ceramic, there was minimal evidence of chemical etching upon exposure to 1 N HCl. Conversely, the surface of the CTP glass ceramic showed significant signs of etching, indicating a higher susceptibility to acid attack. The observed small-sized pits visible in the SEM images likely resulted from the selective dissolution of certain components in the material (e.g., crystalline and/or glassy calcium phosphate phase) during the etching process. **Raman**: The glass ceramics studied in this work consist of different phosphate groups, namely ortho-, pyro-, and meta-phosphate. The peaks assigned to these groups were much sharper for the CTP-F glass -ceramics, suggesting that there were more crystalline phases present. Upon the addition of fluorine, a significant portion of the orthophosphate groups in the glass was utilized for the formation of apatite crystals. These apatite crystals are believed to contain fluorine rather than hydroxyl groups, indicating that the crystal phase is likely fluoro-oxyapatite. This process led to a decrease in the formation of CaTi_4_(PO_4_)_6_ crystals in the glass, and the remaining titanium constituent was partially consumed for the formation of anatase crystals while the rest resided in the glassy phase of the glass-ceramic. In summary, the incorporation of fluorine in the glass ceramics promotes the formation of apatite crystals at the expense of CaTi_4_(PO_4_)_6_ crystals and contributes to the presence of anatase and fluoro-oxyapatite phases. These changes in crystal composition and structure are associated with an improvement in the chemical durability of the glass-ceramic material.
	P	Induced crystallization and physical properties of Li_2_O-CaF_2_-P_2_O_5_: TiO_2_ glass system—Part I. Characterization, spectroscopic and elastic properties (2008) [25]	**XRD**: XRD analysis of the pre-treated glasses confirmed the amorphous nature of the samples. XRD patterns of the glass ceramics after heat treatment at 500 °C exhibited peaks attributed to various lithium phosphate, lithium-titanium-phosphate and titanium phosphate crystal phases. With increasing TiO_2_ content in the glass ceramics, the XRD spectral peaks shifted in intensity and location. **SEM**: SEM images of pre-crystallized samples showed no significant crystallinity, despite the concentration of TiO_2_ within the samples. For crystallized samples, SEM pictures showed an increasing crystallinity with increasing concentration of TiO_2_ up to 0.6 mol%. Upon further addition of TiO2 (0.8 mol%), SEM images showed the presence of larger agglomerated crystalline structures. **DTA**: The DTA curves of the pre-treated and heat-treated samples exhibited significant changes indicating distinct thermal events. The pre-treated sample displayed an endothermic shift attributed to T_g_ occurring between 370 and 400 °C, accompanied by a single exothermic peak associated with crystal growth within the sample. In the heat-treated samples, the endothermic transition for T_g_ also occurred in the same temperature range as observed in the pre-treated sample. However, there were three exothermic peaks (T_c1_, T_c2_, T_c3_) indicating multiple crystal growth processes. Notably, with increasing TiO_2_ concentration up to 0.6 mol% in the glass ceramics, the parameter T_c2_-T_g_ showed a progressive increase, suggesting an enhancement in the glass stability. This finding holds significant scientific impact and implies improved properties for the glass-ceramic material. **Density**: The density of the TiO_2_-free glass ceramic was 2.4495 g/cm^3^. As the concentrations of TiO_2_ increased up to 0.6 mol% the density increased (2.4495–2.4731 g/cm^3^). Upon further addition of TiO_2_ (0.8 mol%), the density decreased (2.4498 g/cm^3^). **Chemical Durability/Weight Loss**: The average dissociation rate (DR) for each sample was calculated using, DR = ΔW/(S × t), where ΔW was the measured weight loss, S was the surface area of the sample and t was the time of immersion. The DR of the TiO_2_-free glass ceramic was 5.37 ((×10^−6^) g/cm^2^/min). With increasing content of TiO_2_ up to 0.6 mol%, the DR decreased to 0.81 ((×10^−6^) g/cm^2^/min), whereafter it increased to 1.72 upon the addition of 0.8 mol% TiO_2_. These results suggested that the addition of TiO_2_ (up to 0.6 mol%) increased the chemical durability of the glass ceramics. This was believed to be due to the titanium ions forming preferentially into tetrahedral configurations, thus participating in the formation of P-O-Ti bonds in the network rather than forming non-bridging oxygens. **FTIR**: As the TiO_2_ concentration in the system gradually increased up to 0.6 mol%, a notable enhancement in the intensity of bands corresponding to TiO_4_ structural units, as well as PO_2_^−^ symmetrical stretching and P-O-P symmetrical stretching was observed by the authors. However, beyond this point (i.e., TiO_2_ concentration up to 0.8 mol%), the intensity of these bands started to decrease. On the other hand, a contrasting trend was observed for bands associated with TiO_6_ structural units and P-O-P asymmetrical stretching. Initially, as the TiO_2_ concentration increased up to 0.6 mol%, the intensity of these bands decreased. However, beyond this point, with a continued rise in TiO_2_ concentration up to 0.8 mol%, the intensity of the TiO_6_-related bands and P-O-P asymmetrical stretching bands exhibited an increase. These findings provide valuable insights into the structural changes induced by varying TiO_2_ concentrations, shedding light on the complex and unpredictable interplay between TiO_2_ and the molecular components in the system. **Raman**: The Raman spectra of the TiO_2_-free glass ceramic revealed three distinctive bands corresponding to symmetric stretching of non-bridging PO_2_ (1068 cm^−1^), symmetric stretching of P-O-P (725 cm^−1^), and bending and torsional vibrations of PO_4_ structural units (400 cm^−1^). Upon introducing TiO_2_ to the glass-ceramic, two additional peaks emerged in the spectra, which were attributed to TiO_6_ units (730 cm^−1^) and isolated TiO_4_ units (900 cm^−1^). As the TiO_2_ content was increased up to 0.6 mol%, the bands associated with symmetrical phosphate units (PO_2_ and P-O-P) and TiO_4_ units exhibited an increase in intensity. Simultaneously, the bands linked to TiO_6_ units and PO_4_ structural units showed a decrease in intensity. Continuing with the addition of TiO_2_ (up to 0.8 mol%), the trend reversed. The spectrum of the sample containing 0.6 mol% TiO_2_ displayed the most prominent intensity for the TiO_4_ band among all the samples, and the band attributed to TiO_6_ units seemed to merge into that of the P-O-P stretching. From these observations, two conclusions can be drawn. Firstly, up to a TiO_2_ concentration of 0.6 mol%, titanium ions favoured forming tetrahedral positions in the glass ceramics. Secondly, the incremental addition of TiO_2_ up to 0.6 mol% possibly promoted the formation of P-O-Ti linkages within the network. These insights provide valuable information on the structural preferences and interactions induced by varying TiO_2_ concentrations, contributing to a deeper understanding of the glass ceramic’s composition and properties. **Elastic and Mechanical Properties**: With increasing TiO_2_ content up to 0.6 mol%, Young’s modulus, shear modulus, Poisson’s ratio, and micro-hardness increased, whereafter these properties were found to decrease upon further addition of TiO_2_ (up to 0.8 mol%).
	P	Induced crystallization and physical properties of Li_2_O-CaF_2_-P_2_O_5_: TiO_2_ glass system—Part II. Electrical, magnetic and optical properties (2008) [68]	**EPR**: EPR spectra showed a strong signal at an approximate g value of 1.940 followed by two weaker signals at g values of 1.965 and 1.971 and a very weak signal at g = 2.001 in all glass ceramics. As the concentration of TiO_2_ increased in the glass ceramics up to 0.6 mol%, the intensities of the signals decreased, whereafter they increased upon further addition of TiO_2_ (0.8 mol%). These observations are consistent with the literature and, as such, indicate that the paramagnetic centers responsible for the observed signals in the EPR spectra are influenced by the concentration of TiO_2_ in the glass ceramics. The strong signal at g ≈ 1.940, along with the two weaker signals at g ≈ 1.965 and 1.971, and the very weak signal at g = 2.001, suggest the presence of distinct paramagnetic species in the material. The initial decrease in signal intensities with increasing TiO_2_ concentration up to 0.6 mol% is in accordance with previous findings reported in the literature. This trend implies that the introduction of TiO_2_ influences the population or stability of the paramagnetic centers, possibly by altering the local chemical environment. However, the subsequent increase in signal intensities upon further addition of TiO_2_ (0.8 mol%) deviates from the previous trend, indicating a non-linear relationship between TiO_2_ content and the paramagnetic centers. This intriguing behaviour might be attributed to the formation of different TiOx species or the interaction between TiO_2_ and other components in the glass ceramics, leading to a more complex paramagnetic response. Overall, the consistency of these observations with existing literature highlights the significance of TiO_2_ in influencing the paramagnetic properties of the glass ceramics. Further investigation and analysis are warranted to precisely elucidate the nature of the paramagnetic centers and their role in determining the material’s properties and potential applications. **UV-Vis, Magnetic, Dielectric Properties**: For readers interested in optical, magnetic, and/or electrical properties, please refer to the appropriate reference [68].
	P	Luminescence spectroscopy of Ti ions in Li_2_O-CaF_2_-P_2_O_5_ glass ceramics (2008) [69]	**XRD**: XRD patterns of the glass ceramics after heat treatment at 500 °C displayed peaks attributed to LiTi_2_(PO_4_)_3_ and TiP_2_O_7_ crystal phases. These results suggest that titanium ions are primarily configured in the Ti^4+^ state in these glass ceramics. The authors state that, based on redox ratio calculations, as the concentration of TiO_2_ increases up to 0.6mol%, the reduction in Ti ions from Ti^4+^ to Ti^3+^ decreases. Upon further addition of TiO_2_ (up to 0.8mol%), this trend reverses. Based on earlier structural studies on these glass ceramics, the authors concluded that Ti^4+^ ions occupy tetrahedral and substitutional octahedral positions in these networks (e.g., TiO_4_), whereas Ti^3+^ ions occupy modifying or octahedral positions (e.g., TiO_6_). When these structural units are formed, they alter the phosphate network, disrupting PO_4_ units and forming P-O-Ti bonds. **UV-Vis, Luminescence Emission**: For readers interested in optical properties, refer to [69].
	P	Preparation of a Calcium Titanium Phosphate Glass-Ceramic with Improved Chemical Durability (2009) [33]	**XRD**: The CPT glass ceramic contains crystalline phases: α-Ca_2_P_2_O_7_, CaTi_4_(PO_4_)^6^, TiO_2_ (anatase), and (TiO)_2_P_2_O_7_. In contrast, the CPT-F glass ceramic has CaTi_4_(PO_4_)_6_ and apatite phases. XRD peaks of CPT-F glass ceramic are sharp and intense. During the formation of crystalline phases in CPT glass-ceramic, titanium is consumed, leaving trace amounts in the residual glassy phase. In CPT-F glass-ceramic, a larger amount of titanium is found in the residual glassy phase, evident from its pale bluish color. Apatite preferentially forms in CPT-F glass due to the decomposition of pyrophosphate groups, promoting the crystallization of apatite and CaTi_4_(PO_4_)^6^ during heat treatment. **Laser Raman Spectroscopy**: There were no significant differences between the wavenumber and intensity of Raman spectral peaks for the CPT and CPT-F glasses. Spectra of both glasses showed peaks attributed to orthophosphate groups (PO_4_^3−^), short-chain phosphate groups (P_2_O_7_^4−^), and long-chain phosphate groups (PO_3_^−^). **EDX**: The nominal compositions (Ca-P-Ti-F in wt.%) of the CPT and CPT-F glasses were determined to be 13.8–18.5–7.7 and 13.4–17.9–7.5–6.0, respectively. The experimental composition of the resulting CPT-F glass was determined to be 13.3–17.8–6.9–2.8, demonstrating the partial loss of fluorine during synthesis (via evaporation). The Ca:Ti:P atomic ratio of the glass surface was determined to be 1.0:3.9:5.1, which is comparable with that of the CaTi_4_(PO_4_)_6_ phase. **SEM**: After treatments in dilute HCl, CPT glass ceramic exhibited a microstructure characteristic of spinodal phase separation, wherein two continuous phases existed. Contrastingly, small-sized pits (<0.1 µm) were seen on the surfaces of the treated CTP-F samples, which could have possibly been due to the dissolution of calcium phosphate phases in the glass ceramics upon treatment with acid. **DTA**: The DTA curves of the CPT and CPT-F glasses show glass transition temperatures at 675 and 615 °C and onset of crystallization peaks at 785 and 685 °C, respectively. The lower temperatures for the CPT-F glass were attributed to the effects of fluorine on thermal properties. **^19^F MAS NMR**: ^19^F MAS NMR analysis was performed on the CTP-F glass and glass-ceramic. For the glass, there was a broad peak in the spectra around −100ppm, whereas in the glass-ceramic, this peak appeared at −103ppm. This peak was believed to be attributed to fluorapatite, thus suggesting that the apatite crystals observed via XRD analysis are that of fluorapatite (e.g., Ca_10_(PO_4_)_6_(O,F_2_)). **ICP-AES (Chemical Durability)**: Upon submerging the CPT and CPT-F glasses and glass ceramics in distilled water, adjusted to pH 7, at 37 °C for 7 days, release curves of Ca^2+^ and P^5+^ ions showed continuous dissolution, except for the CPT-F glass ceramic wherein ionic dissolution was limited. The Ca and P dissolution curves (ion concentrations vs. soaking time) were similar for the glasses and glass ceramics. The enhanced chemical durability for the CPT-F glass ceramics was believed to be due to the effects of fluorine on the crystallization behaviour and microstructure of these samples.
	P	Fabrication and luminescence behaviour of phosphate glass ceramics co-doped with Er^3+^ and Yb^3+^ (2012) [70]	**XRD**: XRD analysis verified the amorphous nature of the precursor glass and identified multiple diffraction peaks for the heat-treated samples, attributed to the LiPO_3_ and TiP_2_O_7_ crystalline phases. Samples were heat treated at temperatures ranging from 450 to 530 °C. The intensity and sharpness of these peaks increased upon increasing the heat-treatment temperature of the samples. **Transmittance Spectra, Luminescence Emission**: For readers interested in optical properties, please refer to the appropriate reference [70].
	P	Synthesis of nanostructured Li_3_Me_2_(PO_4_)_2_F_3_ glass-ceramics (Me = V, Fe, Ti) (2016) [27]	**XRD**: XRD patterns of the TT (Li_3_Ti_2_(PO_4_)_2_F_3_) and TV (Li_3_TiV(PO_4_)_2_F_3_) samples post-synthesis were representative of glasses, with broad humps and the absence of diffraction peaks. Temperature-dependent XRD was performed on the glass precursors to observe the crystallization behaviour at various temperatures. These results suggested that the materials remained amorphous up to approximately 420 °C, whereafter crystallization peaks began to appear on XRD patterns. At least two separate phases were identified in the crystallized samples, one of these being a NAISCON-like Li_3_Fe_2_(PO_4_)_3_ phase. Based on the peak broadening of the XRD patterns, the average grain sizes at 420, 500, and 600 °C were estimated to be approximately 40–60 nm, 75 nm and >100 nm, respectively. The modulation of grain size suggests the potential for enhanced mechanical and functional properties (e.g., chemical durability, surface finish) due to the fine microstructure in the resulting glass ceramics. **DTA**: The temperature ranges corresponding to glass transition (T_g_) and the maximum crystallization peak of the samples were 373 °C to 455 °C and 459 to 514 °C, respectively. T_g_ was relatively lower for Ti-containing samples (lowest for TT and T_g_ of sample TV was lower than that of VV (Li_3_V_2_(PO_4_)_2_F_3_)). **SEM**: SEM images of the samples showed the presence of both sub-100 nm structures and structures of approximately 1 micron in size, which may correspond to the separate and distinct phases identified by XRD analysis. It appeared that the size of grains within a sample increased with increased heat treatment temperatures. **Electrical Conductivity**: For readers interested in additional data pertaining to electrical properties, please refer to the appropriate reference [27].
	P	Syntheses and nanocrystallization of NaF–M_2_O_3_–P_2_O_5_ NASICON-like phosphate glasses (M = V, Ti, Fe) [71]	**XRD**: XRD analysis of the samples prior to heat treatment revealed typical glassy characteristics, with a broad amorphous halo at approximately 2θ = 30° for all compositions. However, some minor reflections were observed, particularly in samples VV (Na_3_V_2_(PO_4_)_2_F_3_) and FF (Na_3_Fe_2_(PO_4_)_2_F_3_), which were attributed to rhombohedral V2O3 and tetragonal VO2, and to hexagonal Na4P2O7 and orthorhombic Fe_3_O_4_, respectively. Additionally, traces of hexagonal Ti_3_PO_2_ were detected in the FT (Na_3_FeTi(PO_4_)_2_F_3_) glass. Upon heat treatment, XRD patterns of samples VV, TT (Na_3_Ti_2_(PO_4_)_2_F_3_), and TV (Na_3_TiV(PO_4_)_2_F_3_) closely matched a reference pattern of Na_3_V_2_(PO_4_)_2_F_3_, indicating that compositions containing Ti_2_O_3_ shared the same crystal structure as the VV compound. The absence of explicit identification of Ti-containing phases does not imply their absence; rather, it suggests that Ti may substitute other transition metals, resulting in isostructural phases which emphasizes the complexity of crystallization behaviour in these materials. **DTA**: The DTA traces of glass samples prior to heat treatment consisted of a glass transition step in the 365 to 481 °C range (temperatures varied depending on glass composition) followed by two or three crystallization peaks at temperatures ranging from 400 to 557 °C. It is believed, based on results from XRD analysis, that the main crystallization peak can be attributed to NASICON phase. **Grain size**: The average grain size in the samples post-crystallization ranged from 63 to 112 nm. Larger grains were identified in samples VV, TT, TV and smaller grains were in samples FF, FV (Na_3_FeV(PO_4_)_2_F_3_), and FT. It appeared the grain size was correlated to the NASICON phase purity, wherein samples with highest NASICON phase purity (VV, TT, TV) had larger grains. **FESEM**: SEM images of the heat-treated samples showed the presence of numerous ca. 100 nm and below grains. The grains in samples VV and TV appeared to maintain contact with each other in a continuous phase, whereas grains in the remaining samples were separated from each other in discontinuous phases with the presence of pits and crevices. **Electrical properties**: For readers interested in electrical properties, please refer to the appropriate reference [71].
	Si	Influence of TiO_2_ content on phase evolution, microstructure and properties of fluorcanasite glass-ceramics prepared through sintering procedure for dental restoration applications (2018) [72]	**XRD**: Heat-treated samples FC0, FC6, and FC9 exhibited peaks attributed to calcium fluoride and fluorcanasite crystals. These crystals were also present in the sample FC12, in addition to goetzenite (Na_0_._9_Ca_2_._6_Ti_0_._5_Si_2_O_7_._1_(OH)_0_._9_F). When the crystallization temperature of sample FC12 was increased to that of the second crystallization peak (observed via DTA analysis), titanite was detected as the only crystalline phase present. **DTA**: The DTA curves of the samples showed one exothermic peak at temperatures ranging from 736 to 750 °C and one endothermic peak at temperatures ranging from 1000 to 1046 °C. These peaks can be attributed to the occurrence of crystallization and the dissolution of these crystalline phases into the glass matrix, respectively. In sample FC12, there was an additional exothermic peak present. The change in glass transition temperature (T_g_) and dilometric softening point temperature (T_d_) between samples suggested that these values were significantly increased (534 to 573 °C and 580 to 623 °C) by the increasing content of TiO_2_ in the glass, thus increasing glass viscosity and decreasing the occurrence of crystallization. **FESEM-EDX**: A glass-ceramic material, FC6, with increased TiO_2_ content (up to 6 weight ratio) exhibits a homogeneous distribution of dark grey interlocked crystals with blade-like morphology alongside light grey spheroid crystals. These crystals were identified as fluorcanasite and calcium fluoride, respectively, based on EDX analysis. For sample FC0, FESEM images showed a homogenous distribution of blade-like interlocking crystals mixed with spheroid crystals. Based on EDX analysis, these crystals were believed to correspond to fluorcanasite and calcium fluorine, respectively. The microstructure of FC6 shows evidence of coarsening, with larger fluorcanasite crystals compared to the FC0 glass-ceramic. This size increase was attributed to higher glass viscosity and reduced crystallinity. In FC9, calcium fluoride crystals remain homogeneously dispersed, but the interlocking arrangement between fluorcanasite crystals declined due to decreased crystallinity. Needle-like goetzenite crystals were also observed in FC9. In FC12, both needle-like and spheroid dark grey crystals were widely dispersed as the dominant crystalline phase and the interlocked crystal arrangement was not detectable. XRD patterns of FC12 confirmed the presence of goetzenite crystals. In summary, the varying TiO_2_ content in these glass ceramics influences the crystal morphology, distribution, and crystallinity, leading to different microstructural features observed in each composition. **Chemical Solubility**: The chemical solubility increased upon adding TiO_2_ to the base glass up to 6 weight ratio (FC6), whereafter it decreased significantly. The decreased chemical solubility in samples FC9 and FC12 were attributed to the addition of TiO_2_ as TiO_2_ increased the structural connectivity of the glass network, thus increasing the chemical stability of the residual glass phase. **Mechanical Properties**: With increasing TiO_2_ content in the glass ceramics, the flexural strength and the fracture toughness decreased as a result of the decreased crystallinity and suppressed interlocking arrangement of crystals from FC0 to FC12. On the contrary, the Vickers micro-hardness increased with increasing TiO_2_ content, which was believed to be due to the precipitation of harder crystalline phases and the improved sinterability that occurred upon adding TiO_2_ to the glass matrix.
	Si	Effect of complex nucleation agents on preparation and crystallization of CaO-MgO-Al_2_O_3_-SiO_2_ glass-ceramics for float process (2016) [26]	**XRD**: Glass samples treated at 700 °C for 30 min show that the T (9CaO·6MgO·21Al_2_O_3_·49SiO_2_·4K_2_O·4Na_2_O·2ZnO_2_·5TiO_2_), TP (T·2P_2_O_5_), and TFP (T·2CaF_2_·2P_2_O_5_) samples exhibit broad scattering peaks, indicating no crystal precipitation in the glass matrix. However, the TF (T·2CaF_2_) sample exhibits tiny diffraction peaks corresponding to the diopside crystal phase near 30° and 35 °C. For glass-ceramic samples treated at 730 °C for 30 min followed by 930 °C for 30 min, the T, TF, TP, and TFP samples all have the same major crystalline phase, which is diopside (Ca(Mg,Al)(Si,Al)_2_O_6_). This indicates that the addition of CaF_2_ and P_2_O_5_ does not significantly affect the major crystalline phase. Furthermore, the presence of 2 wt.% CaF_2_ in the glass ceramic leads to the precipitation of diopside as the major crystal phase, along with the observation of fluorpargasite and pargasite phases. In the TFP sample, compared to the TF sample, there is an increase in the content of pargasite and fluorpargasite, while the content of diopside decreases. In summary, the heat treatment of the glass and glass-ceramic samples results in varying crystalline phases, and the addition of CaF_2_ and P_2_O_5_ influences the content and distribution of specific crystalline phases in the glass-ceramic materials. **Raman**: The bands located in the 800–1250 cm^−1^ range are assigned to Si-O stretch vibrations of Qn tetrahedral units, where *n* = 1,2,3,4. The content of Q^1^, Q^2^, Q^3^, and Q^4^ for the sample T is 9.54%, 21.97%, 49.29%, and 19.20%, respectively. These relative fractions suggest that the network is formed mainly by the SiO_3_^2−^ chain, Si_2_O_5_^2−^ sheet, and SiO_2_ three-dimensional network. With the addition of CaF_2_ to sample TF, the content of Q2 increased to 24.59% and the content of Q4 decreased to 15.26%, suggesting that fluorine decreased the network connectivity by replacing the bridging oxygens in =Si-O-Si= with weaker =Si-F linkages. **DSC**: For sample T, the practical melting temperature and forming temperature were approximately 1489 °C and 1286 °C, respectively. When 2 wt.% CaF_2_ was added, the practical melting temperature and forming temperature of TF specimen decreased to around 1463 °C and 1260 °C, respectively. Small endothermic peaks (T_g_) and a more intense exothermic peak (crystallization, T_p_) were observed for all materials. The addition of CaF_2_ (sample TF) decreased glass viscosity, as well as the practical melting and forming temperatures, compared to the CaF_2_-free sample (sample T). Furthermore, the glass transition temperature (T_g_) and the crystallization peak temperature (T_p_) were decreased for sample TF (698 and 880 °C) compared to sample T (668 and 875 °C). **FESEM**: FESEM images of the T glass sample treated at 700 °C for 30 min showed some phase-separated droplets of approximately 150 nm in size, throughout the amorphous matrix. When 2 wt.% CaF_2_ and 2 wt.% P_2_O_5_ were added to the base glass (sample T), the droplets grew slightly to approximately 200 nm in size and were more dispersed in the matrix. In the FESEM images of the CaF_2_-containing glass (sample TF), similar nucleated particles of approximately 200 nm in size were observed, but they appeared to have developed from the continuous matrix rather than the droplet phase. **SEM**: SEM images of the T glass-ceramic sample treated at 730 °C for 30 min followed by 930 °C for 30 min showed some granular crystals of approximately 1 µm in size distributed irregularly in the matrix. With the addition of 2 wt.% CaF_2_ (sample TF), the crystal size decreased to 150 nm and the number of grains increased, thus indicating an increased crystallinity in the TF sample compared to the T sample. When 2 wt.% CaF_2_ and 2 wt.% P_2_O_5_ were added (sample TFP), disk-shaped crystals of approximately 0.4 µm were observed and appeared to be separated by smaller needle-like crystals.
	Si	Crystallization and microstructure of CaO-MgO-Al_2_O_3_-SiO_2_ glass-ceramics containing complex nucleation agents (2014) [30]	**XRD**: Samples F (14CaO·10MgO·12Al_2_O_3_·50SiO_2_·2K_2_O·3Na_2_O·1SbO_3_·8CaF_2_), FT (F·3TiO_2_), and FZ (F·3ZrO_2_) exhibit similar XRD patterns, with peaks corresponding to primarily diopside and anorthite crystalline phases. These results suggest that the addition of TiO_2_ to the base glass did not change the types of crystalline phases precipitated. **DTA**: The Ti-containing FT sample showed a prominent exothermic peak at 919 °C, which was attributed to the formation of crystalline phases (e.g., diopside and anorthite). The temperature corresponding to this peak decreased with the addition of TiO_2_, suggesting that a complex nucleating agent, containing both fluorine and titanium for example, can increase the occurrence of crystallization in CMAS glasses. **SEM**: SEM images of the F sample showed a disorderly arrangement of diopside and anorthite crystals mixed with glass phases whereas images of the Ti-containing FT sample showed the presence of large, distinct, rod-like crystals and high crystallinity. **Chemical Solubility**: The chemical solubility of samples F and FZ was not assessed. Comparing the chemical resistance of samples FT and FP (F·3P_2_O_5_), sample FT exhibited higher resistance. However, sample FP had a higher crystallization ratio. The Vickers hardness of FT was greater than FP due to the higher hardness of the main crystalline phase, diopside, in sample FT compared to the main crystalline phase, pyroxene, in sample FP. These differences in chemical resistance, crystallization ratio, and Vickers hardness between samples FT and FP can be attributed to their varying crystalline phases and microstructures and highlight the criticality of understanding composition–structure–property relationships in these materials.
Mixed Primary Glass Formers	Si/P	The effect of TiO_2_ concentration on properties of apatite-mullite glass-ceramics for dental use (2016) [73]	**XRD**: XRD analysis confirmed the amorphous nature of the pre-treated glasses. XRD patterns of the heat-treated glasses exhibited diffraction peaks attributed to primarily fluorapatite (Ca_5_(PO_4_)_3_F) and mullite (Al_6_Si_2_O_13_) crystalline phases, as well one phase of cristabolite. The addition of TiO_2_ to the glasses had no effect on the types of crystalline phases precipitated. **DTA**: Data showed glass transition at temperatures (T_g_) ranging from 650 to 730 °C, a first crystallization peak at temperatures ranging from 865 to 920 °C, and a second crystallization peak at temperatures ranging from 970 and 1070 °C. The first and second crystallization peaks were attributed to the precipitation of fluorapatite and mullite, respectively, as confirmed by XRD. These crystallization peaks, most notably the second peaks, were more defined for samples containing high concentrations of TiO_2_, suggesting the occurrence of bulk crystallization in these samples. Furthermore, with increasing TiO_2_ content in the glasses, the temperatures corresponding to T_g_ and crystallization peaks, as well as the viscosity of the glass samples, decreased. **SEM-EDX**: With increasing TiO_2_ content in the glass ceramics, SEM and EDS analysis showed an increased crystal grain size, a more refined microstructure, and an increased number of crystals (apatite and mullite phases) compared to samples with low TiO_2_ content. **XRF**: The raw data from XRF analyses showed inconsistencies with the discussion presented by Fathi and Johnson. For example, when comparing the wt.% of TiO_2_ after melting and before melting, authors stated that TiO_2_ content was found to increase after melting in all compositions. However, based on the raw XRF data, TiO_2_ decreased after melting in samples HG1T2.5, HG1T3, and HG1T3.5. Based on this ambiguity, the XRF data were not considered further in this review. **Chemical Solubility**: The HG1T2 formulation (2 wt.% TiO_2_) had the highest solubility among the tested materials, while the HG1T2.5 (2.5 wt.% TiO_2_) composition had the lowest solubility. Based on the lack of significant differences between many of the chemical solubility values of the glass ceramics, the effect of increasing TiO_2_ content on the chemical solubility of these samples is somewhat ambiguous. However, directionally, it appears that the solubility increased as the TiO_2_ content increased from HG1T0.0 to HG1T2, but decreased thereafter for the HG1T2.5 composition. Adding 3 wt.% TiO_2_ then increased the solubility, but further addition of TiO_2_ up to 4 wt.% decreased solubility. Finally, adding 4.5 and 5 wt.% TiO2 increased solubility again; however, this increase was not statistically significant in comparison to the chemical solubility of sample HG1T4. **Mechanical Properties**: The highest biaxial flexural strength (BFS) was achieved in the material with 1.5 wt.% TiO_2_. Increasing the TiO_2_ content led to a strengthening effect in the following order: HG1T0.0 < HG1T0.5 < HG1T1 < HG1T1.5. However, the BFS value decreased in the material containing 2 wt.% TiO_2_ (HG1T2). Upon further increasing the TiO_2_ content up to 2.5 wt.%, the highest BFS was observed again, followed by a decrease in strength values with 4 wt.% TiO_2_. Interestingly, BFS values increased again with 4.5 and 5 wt.% TiO_2_. The non-monotonic nature of the data signals the need for a more in-depth investigations, acknowledging the intricate interactions of factors and the potential for unexpected or counterintuitive outcomes.
	Si/B	Influence of TiO_2_ content on the crystallization and microstructure of machinable glass-ceramics (2016) [31]	**XRD**: The predominant crystalline phase present in all samples was fluorophlogopite mica, KMg_3_(AlSi_3_O_10_)F_2_. Additionally, forsterite (Mg_2_SiO_4_) and cordierite (Mg_2_Al_4_Si_5_O_18_) appeared as minor or secondary crystalline phases. It was reported that the intensity and number of these crystalline phases increased as the heat treatment temperature increased from 800 °C to 1080 °C. The same effect on the crystalline phases was observed as the content of TiO_2_ increased from 1 to 10 wt.% under the same heat treatment temperature. **DSC**: Increasing the content of TiO_2_ from 1 to 10 wt.% caused an increase in glass transition temperature (T_g_) and both crystallization peak temperatures (T_p_^I^ and T_p_^II^). For sample MGT-1, MGT-2, and MGT-3, the T_g_ was 705 °C, 708 °C, and 723 °C, respectively, while the exothermic crystallization peak temperatures were 874 °C (T_p_^I^) and 938 °C (T_p_^II^), 882 °C (T_p_^I^) and 959 °C (T_p_^II^), 902 °C (T_p_^I^) and 980 °C (T_p_^II^), respectively. **FTIR**: The FTIR spectra of the samples displayed a peak at 472 cm^−1^, corresponding to Si-O-Si bending vibrations of [SiO_4_] units and Ti-O-Ti stretching vibrations. Within the 660–690 cm^−1^ region, bands were identified as vibrations in [TiO_4_] tetrahedra. In all four samples, peaks at 685 cm^−1^ and 721 cm^−1^ were attributed to the stretching vibrations of O-Si-O in [SiO_4_] tetrahedra and Si-O-Ti between [SiO_4_] and [TiO_4_] units within the network. Notably, these bands shifted towards higher wavenumbers with an increase in TiO_2_ content. **SEM**: With increasing TiO_2_ content in the glass ceramics, the aspect ratio of fluorophlogopite mica crystals present in the glass matrix increased. Specifically, as TiO_2_ was added to the matrix, the microstructure became increasingly uniform, dense, and blocky. At the maximum TiO_2_ content (10 wt.%), the microstructure was made of predominantly interconnected, large, blocky fluorophlogopite mica crystals, which have the tendency to prevent crack formation and growth. **Mechanical Properties**: With increasing heat treatment temperatures of the glass ceramics, the Vicker’s hardness values (Hv) decreased. With increasing content of TiO_2_ (1–10 wt.%), the Hv values decreased, and the machinability of the samples increased. The decreased hardness was believed to be correlated to the formation of the interlocking, dense, blocky microstructure, as evidenced by SEM.
	Si/P	On the microstructure of biocomposites sintered from Ti,HA and bioactive glass [74]	**XRD**: Diffraction peaks for the precipitated phase in the bioactive glass after heat-treating at 800 °C for 2 h were identical to those of hydroxyapatite. **Density**: Density analysis was performed on the biocomposite only (HA powder + Ti powder + BG), therefore there is no information available on the specific roles of Ti and/or F within the glass component of the biocomposite. **TEM-EDS**: TEM-EDS analysis were performed on the biocomposite only (HA powder + Ti powder + BG), therefore there is no information available on the specific roles of Ti and/or F within the glass component of the biocomposite.

**Table 7:** Trends in a selection of experimental findings from articles investigating glass materials.

	Coordination Number of Network Forming Element	Density and Network Connectivity	Glass Transition Temperature	Mechanical Characteristics	Chemical Durability	Biological Responses
Overall trends associated with increasing Ti content	Increases ^a,b^ [18,19]	Increases ^d,a,b^ [16,18,19]	Increases ^a,b^ [18,19]	Increases Young’s, shear, bulk modulus, decreases microhardness and Poisson’s ratio ^d^ [16]	Not addressed	Not addressed
	Decreases ^c,c,c^ [14,65,66]	Decreases ^c,d,c^ [14,15,65]	Decreases ^d^ [15]			
Overall trends associated with increasing F content	Decreases [65,66]	Not addressed	Not addressed	Not addressed	Not addressed	Not addressed

^a^ = phosphate. ^b^ = borosilicate. ^c^ = borate. ^d^ = borophosphate.

**Table 8:** Overall trends in a selection of experimental findings from articles investigating glass ceramics.

	Coordination Number of Network Forming Element	Densit and Network Connectivity	Glass Transition Temperature	Mechanical Characteristics	Chemical Durability	Biological Responses
Overall trends associated with increasing Ti content	Not addressed	Increases ^a^ [25]	Increases ^a,b,c^ [25,31] *, [72] * Decreases ^a,d^ [27] *, [73] *	Increases Young’s modulus, shear modulus, Poisson’s ratio, and micro-hardness (up to 0.6 mol% TiO_2_) ^a^ [25] Increases viscosity ^c^ [72] Decreases viscosity ^d^ [73] Increases microhardness ^c^ [72] Decreases microhardness ^b^ [31] Decreases fracture toughness and flexural strength ^c^ [72]	Increases ^a^ [25], Increases (0–6 wt. ratio TiO_2_) and Decreases (6–12 wt. ratio TiO_2_) ^c^ [72]	Not addressed
Overall trends associated with increasing F content	Decreases ^c^ [26] *	Not addressed	Decreases ^c,a^ [26] *, [33] *	Decreases viscosity ^c^ [26] *	Increases ^a,a^ [32,33]	Not addressed

^a^ = phosphate. ^b^ = borosilicate. ^c^ = silicate. ^d^ = phosphosilicate. An asterisk indicates that the finding was reported for a glass-ceramic precursor material (prior to heat treatment).

## Data Availability

No new data were created or analyzed in this study. Data sharing is not applicable to this article.
